# Healthy Food Service Guidelines for Worksites and Institutions: A Scoping Review

**DOI:** 10.3390/ijerph22081194

**Published:** 2025-07-30

**Authors:** Jane Dai, Reena Oza-Frank, Amy Lowry-Warnock, Bethany D. Williams, Meghan Murphy, Alla Hill, Jessi Silverman

**Affiliations:** 1 Department of Health Systems and Population Health, University of Washington, Seattle, WA 98195, USA; 2 Division of Nutrition, Physical Activity, and Obesity, National Center for Chronic Disease Prevention and Health Promotion, Centers for Disease Control and Prevention, Atlanta, GA 30341, USA; 3 Center for Science in the Public Interest, Washington, DC 20005, USA; 4 Chronic Disease and Health Equity Unit, County of San Diego Health and Human Services Agency, San Diego, CA 92101, USA

**Keywords:** food service guidelines, nutrition, health policy, evaluation, food systems

## Abstract

Healthy food service guidelines (HFSG) comprise food, nutrition, behavioral design, and other standards to guide the purchasing, preparation, and offering of foods and beverages in worksites and institutional food service. To date, there have been few attempts to synthesize evidence for HFSG effectiveness in non-K-12 or early childhood education sectors, particularly at worksites and institutional food services. We conducted a scoping review to achieve the following: (1) characterize the existing literature on the effectiveness of HFSG for improving the institution’s food environment, financial outcomes, and consumers’ diet quality and health, and (2) identify gaps in the literature. The initial search in PubMed and Web of Science retrieved 10,358 articles; after screening and snowball searching, 68 articles were included for analysis. Studies varied in terms of HFSG implementation settings, venues, and outcomes in both U.S. (*n* = 34) and non-U.S. (*n* = 34) contexts. The majority of HFSG interventions occurred in venues where food is sold (e.g., worksite cafeterias, vending machines). A diversity of HFSG terminology and measurement tools demonstrates the literature’s breadth. Literature gaps include quasi-experimental study designs, as well as interventions in settings that serve dependent populations (e.g., universities, elderly feeding programs, and prisons).

## 1. Introduction

Healthy food service guidelines (HFSG) provide parameters for organizations to serve or sell foods and beverages that contribute to healthy diets. HFSG typically use standards for procuring, offering, and/or promoting healthier foods and beverages in food service operations in worksites and community settings [1]. HFSG may be adopted by individual institutions (e.g., hospitals, universities), other employers for onsite food service venues, or governments for public food procurement and food service activities (e.g., correctional facilities, public feeding programs, concessions, and vending on government property) [2,3]. HFSG can be tailored for venues where food is sold (such as an employee cafeteria where consumers choose from a variety of foods and beverages) and venues where food is served (such as a senior center that serves congregate meals at subsidized or no cost, with limited or no consumer choice) [4]. Although recommended standards vary based on setting and other factors, best practices for HFSG include aligning with authoritative, evidence-based dietary guidance (such as the Dietary Guidelines for Americans (DGA) in the United States (U.S.)) and addressing both foods and nutrients to encourage (fruits, vegetables, whole grains) and those to limit (added sugars, fried foods, sodium) [3,5]. HFSG may also include (1) behavioral strategies to encourage healthier choices through product placement, pricing, and promotion, or (2) standards to advance other goals such as energy efficiency and limiting food waste—to reduce the environmental footprint of the food service operation.

Common HFSG goals include promoting better diet quality and health of consumers, enabling employers and community settings to model healthier food and beverage availability, and using such institutions’ purchasing power to foster greater availability of healthier products throughout the food supply. The World Health Organization developed an action framework for healthy public food procurement and service policies, which provides principles of healthy diets, guidance, and country examples to inform policy development and implementation [3]. Countries that have adopted HFSG for government food procurement and catering include Brazil and the United Kingdom [6,7]. In the U.S., several national HFSG models have been developed, including the Food Service Guidelines for Federal Facilities, the Good Food Purchasing Program, and the National Alliance for Nutrition and Activity Model Nutrition Standards for Grab-and-Go Foods and Beverages [4,8,9,10]. Subnational governments in the U.S., including the states of Washington and Vermont, New York City, and San Diego County, have also developed HFSG standards that are informed by national models but tailored to their context and food service activities [11,12,13,14].

To our knowledge, attempts to synthesize the evidence on the effectiveness of healthy food procurement and service policies in any setting have primarily examined school-based findings [15,16]. These studies found that nutrition standards in school settings have led to improvements in the food environment [17], diet quality [18], and weight-related outcomes [19]. However, these findings are not necessarily generalizable to other populations or settings. The peer-reviewed evidence for the effectiveness of HFSG in non-school settings has not been scoped or synthesized in the last decade [15], so current evidence gaps are not widely understood and remain unaddressed. With growing interest in HFSG at the local [20], state [21], and national [22] levels, addressing gaps in the literature regarding benefits of HFSG can support more widespread adoption and inform implementation.

Whereas a systematic review is used to synthesize a body of evidence and draw conclusions about the effectiveness of an intervention and the implications for policy or practice, a scoping review is indicated when the investigators’ goals are to assess the extent of evidence on a topic, characterize how research is conducted, and identify knowledge gaps [23]. Therefore, the objectives of this scoping review are to (1) characterize the scope of evidence on the effectiveness of HFSG for improving the food environment, diet quality, and health outcomes in (non-school) worksites and institutional settings, and (2) identify critical gaps in this body of evidence to inform future research.

## 2. Methods

### 2.1. Data Sources and Search Strategy

This scoping review was conducted in accordance with PRISMA-ScR guidelines [24]. Author JS drafted the scoping review protocol a priori. It was further revised for feasibility and scope by the research team. Staff at the Center for Science in the Public Interest and members of the Nutrition and Obesity Policy Research and Evaluation Network’s Food Service Guidelines Work Group provided additional feedback. The protocol was finalized in June 2022 and is available upon request.

We performed a systematic search of the scientific literature in July 2022 to identify studies relevant to the review objectives. One author (JD) and a Washington State University health science librarian developed a search strategy to identify relevant literature from two research databases: PubMed and Web of Science. We selected these databases because they include disciplines of public health nutrition, psychology, and behavioral economics from which we anticipated reviewing research studies. The protocol defined three primary queries for constructing the search strategy: modifiable elements of HFSG, HFSG setting and venue, definitions of HFSG, and healthy eating frameworks. The complete list of search terms and their order of operations is provided in Supplemental Tables S1 and S2. Search terms were intentionally broad to ensure HFSG was widely captured, as interventions are highly variable in the ways they are defined, studied, and presented.

### 2.2. Inclusion Criteria

We used six inclusion criteria for this review of peer-reviewed publications. First, papers were written or published in English. Second, papers reported on an original study and/or evaluation of an HFSG intervention, which we defined as establishing guidelines or standards for the nutritional quality of foods and/or beverages served or sold in worksites, government programs or facilities, or institutional settings, including one or more measures to increase the provision of healthier foods and nutrients (e.g., fruits, vegetables, and whole grains) or limit the provision of less healthy foods and nutrients (e.g., fried foods, sugary drinks, and sodium). Third, papers examined worksites or institutions (e.g., government programs or facilities and institutional or community settings). Fourth, the research either directly measured or simulated at least one of the following dependent variables: changes to the food environment, consumer behavior, diet quality, and/or health. We defined changes to these variables in two ways: change over time (measured or estimated over at least two points in time) or change by comparison (a point-in-time assessment between intervention and control groups). Fifth, the research targeted adults or children aged five years and older (not in school or early childcare and education (ECE) settings, with the exception of after-school or out-of-school time). And sixth, papers were published in 1997 or later. After completing the initial search, we decided to limit inclusion to papers published in 2012 or later, based on when the authors of a 2014 systematic review of healthy food procurement interventions ended their search [15]. We were agnostic to the type of healthy guidelines or standards applied to different intervention studies; for example, we included papers that assessed school-based nutrition guidelines that were adopted in hospital cafeterias.

Based on a limited initial search, we expected a wide range of study designs to assess the effects of HFSG on our outcomes of interest. Because this review aimed to scope all relevant literature rather than synthesize the evidence, we did not exclude studies based on study design. Therefore, we considered experimental, quasi-experimental, observational, and simulation studies. Qualitative studies were also eligible for consideration if they met the inclusion criteria.

In summary, we included studies concerning food service venues in worksites, health care facilities (excluding therapeutic and medically tailored meals), correctional facilities, government programs, recreation centers, food pantries, and other public or institutional settings. We excluded studies that exclusively concerned freestanding restaurants and commercial food service venues. We excluded studies that focused on breakfast and lunch meal programs in K-12 school and ECE settings, as nutrition standards in these programs have been previously studied and synthesized in the literature [25,26,27].

### 2.3. Citation Review Process

**Figure 1:**
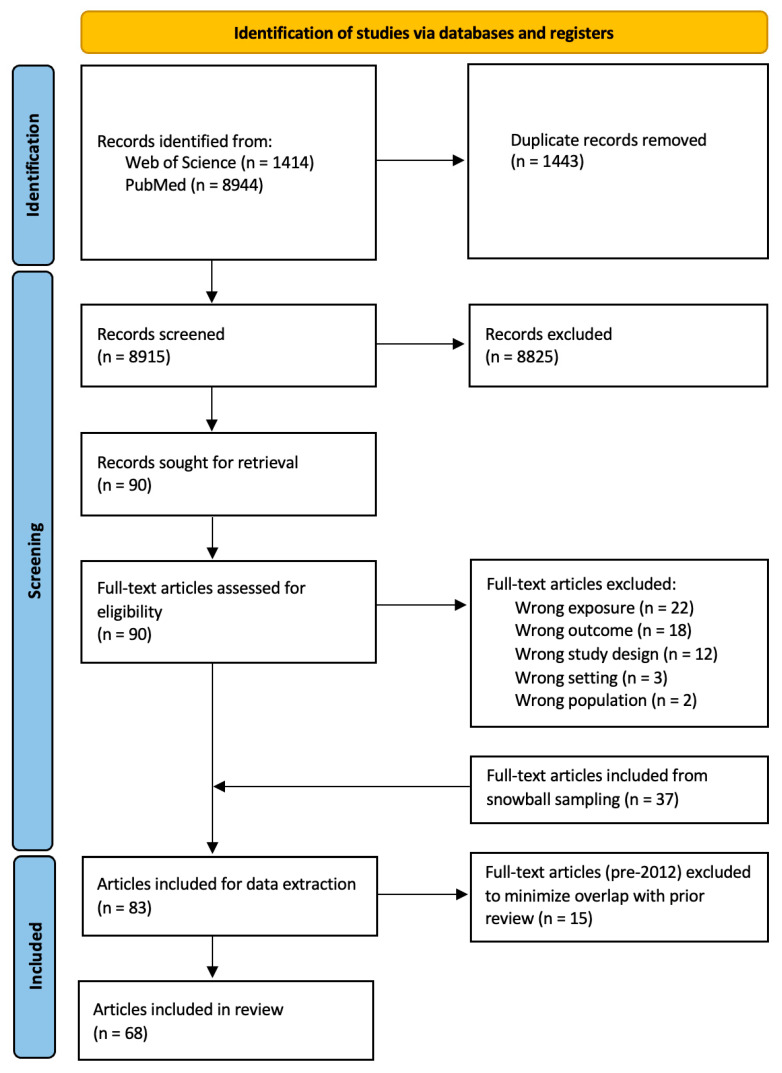
PRISMA ScR flow diagram of article inclusion for analysis in scoping review. Note: At full-text screening, some full-text articles were excluded for failing to meet multiple inclusion criteria.

The citation review process is presented in a PRISMA-ScR flow chart (Figure 1). The research team used Rayyan.ai to collate search results and document the entire screening and study selection process [28]. The initial search strategy returned 10,358 citations from the two selected databases. Rayyan’s auto-detect feature removed 1443 duplicate citations. Two co-authors (JD and JS) conducted pilot screening for inclusion at the title and abstract level until they reached strong agreement (raw agreement > 98%, kappa > 0.80, *n* = 700). One coder (JD) then completed the remainder of the title and abstract screening. After this stage, 90 citations were included for full-text review. For full-text screening, the same two coders (JD and JS) had to independently vote “yes” to include a study for analysis. In the case of disagreement, the two coders would reconcile. If there was no consensus, a third coder (AH) provided input. After full-text review, 46 studies met all inclusion criteria. JD conducted snowball searches from included studies’ references to identify additional citations not retrieved by the broad search strategy; after full-text screening (JD, JS), additional references (*n* = 36) were included. One additional paper was informally identified during review. Finally, papers before 2012 (*n* = 15) were removed, as noted above, to minimize overlap with one prior systematic review [15].

Studies included through the initial, broad search had minimal overlap with studies identified and included through snowball searching regarding keyword, title, and abstract content (see Supplemental Table S3 and Figures S1 and S2). The final analytical sample included 68 studies.

### 2.4. Data Extraction and Synthesis

JD created a standardized extraction tool in Google Forms to facilitate systematic data extraction from the final sample of 68 studies. JD recorded from each study: authors’ names, publication year, study geographic location, study setting (e.g., worksite and hospital cafeteria), study venue (e.g., where foods are sold and where foods are served), details of the intervention (e.g., objectives, independent variables, duration, dosage, and components related and unrelated to HFSG), details of outcomes of interest (e.g., specific measures), study population, sample size, study design, notable findings, and acknowledged limitations. JS independently verified the extracted data for accuracy and completeness.

To tabulate the data, we grouped studies by reported outcomes of interest: food environment, consumer behavior, diet quality, health, and financial implications. The first four outcomes were specified a priori. These outcomes correspond with our conceptual framework of HFSG intervention impact—in which changes to the food environment are expected to influence consumer behavior and diet quality, which in turn influence health outcomes. The fifth reported outcome (financial implications) was identified after completing the full-text review as an important, discrete outcome of FSG interventions that would be of interest to institutional decision-makers. The conceptual framework and the specified outcome categories are adapted from key impacts of HFSG identified by global health authorities and prior publications [2,3,15].

We defined five outcome categories. Food environment outcomes measured the availability of healthier or less healthy items being served or offered for sale (e.g., modification of ingredients or recipes, addition of menu items, making healthier choices the default). Consumer behavior outcomes measured consumers’ selections or purchases (distinct from consumption) of healthier or less healthy offerings in venues where they had multiple options. Diet quality outcomes measured consumption of healthier or less healthy items; intake of key food groups, nutrients, or food components (e.g., vegetables, fiber, and sodium); or alignment with a holistic diet quality index. Health outcomes measured anthropometric or biochemical indicators of diet-related chronic disease risk (e.g., weight and blood pressure) and diet-related chronic disease morbidity or mortality (e.g., projected incidence of diabetes). Financial implications, distinct from consumer behavior, measured the short- and long-term financial consequences associated with the HFSG intervention (e.g., implementation costs, changes in overall revenue of the food service operation, and projected health care cost savings).

In this analysis, we organized studies by whether they were conducted in or outside the U.S.—a decision driven by our affiliations with U.S. institutions and collective expertise in U.S. food systems. Notably, more included studies were conducted in the U.S. than in any other country (*n* = 34). However, we recognize that the diversity of researchers and research methodologies means that studies conducted outside the U.S. are not directly comparable to one another, nor are they necessarily representative of work conducted outside the U.S. Therefore, we consider these non-U.S. studies as providing information on the types of study designs, approaches, and outcomes that U.S. studies may consider in future HFSG research.

## 3. Results

### 3.1. Description of Included Studies

This scoping review included 68 published papers, with 50% conducted in the U.S. and 50% conducted outside the U.S. (the latter henceforth called “non-U.S.” studies). While we consider each paper a stand-alone study, there were 61 unique HFSG interventions among the included studies. Of the remaining seven, one was a follow-up study to assess long-term impacts of an initial HFSG intervention [29], and six published a different process or outcome from the same HFSG intervention [30,31,32,33,34,35]. Descriptive information on all included studies is summarized in Table 1. Individual studies often took place in multiple intervention settings and venues, and many studies assessed multiple outcomes.

#### Comparison of U.S. Versus Non-U.S.-Based Studies

One key distinction in study characteristics between studies in U.S. and non-U.S. settings is the prevalence of different study designs. In the U.S., only 6% of studies used a randomized controlled trial (RCT) to evaluate HFSG intervention outcomes, while 18% of non-U.S. studies used RCT designs. Most U.S. studies (53%) used a pre-post without control design compared to a smaller proportion of non-U.S. studies (38%).

Across all studies, 57% assessed the food environment, 34% assessed consumer behavior, 34% assessed financial implications, 24% assessed diet quality, and 9% assessed health outcomes.

**Figure 2:**
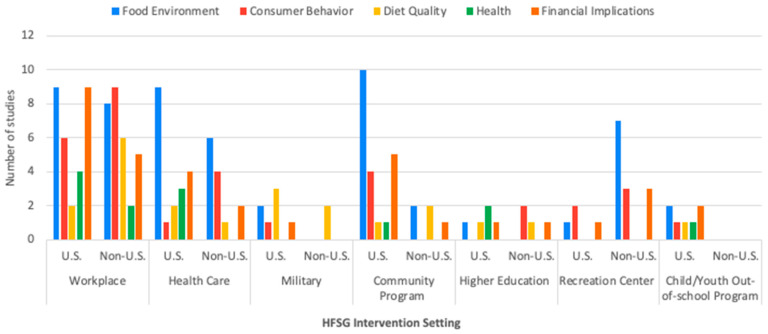
Number of included studies (N = 68) reporting outcomes in each category by healthy food service guidelines (HFSG) intervention setting and whether this study was conducted in the U.S. or outside of the U.S. (non-U.S.). Samples may sum up to more than 68, as some studies assessed multiple HFSG intervention settings and HFSG outcome types.

We identified seven distinct settings in which HFSG were applied across the final sample. Worksites include food service settings that primarily serve employees who work onsite. Health care settings include food service operations in hospitals and other facilities where individuals go to receive health care. Military settings include dining facilities located in or adjacent to military housing or training facilities that serve members of the armed forces or their family members. Community programs include congregate meal programs, public zoo facilities, parks, and other food service venues in institutions or programs that serve the public. Higher education settings include food service operations serving post-secondary education institutions, including colleges and universities. Recreation centers include food service operations in recreational and sport facilities. Child and youth out-of-school time programs included food service integrated into after-school programs and other activities for children and youth outside of the formal school day. U.S. studies most frequently assessed HFSG in worksite (50%), health care (38%), and community program (38%) settings, while non-U.S. studies assessed HFSG primarily in worksite (56%), health care (29%), and recreation center (24%) settings. Higher education and recreation center settings were the two intervention settings least frequently assessed in U.S. studies (9% each). And in non-U.S. studies, higher education and child/youth out-of-school time settings were least frequently assessed (6% and 0%, respectively). Many studies also included similar objectives for HFSG interventions to improve the food supply—such as reducing sodium, portion size, and sugary-beverage consumption, and increasing healthy snack availability. Figure 2 shows the distribution of the five different HFSG outcome types across HFSG intervention settings in U.S. and non-U.S. studies.

The majority (88%) of HFSG interventions occurred in venues where foods were sold. In all studies, these venues were primarily cafeterias, canteens, or dining halls (56% U.S. and 65% non-U.S.) and vending machines (44% U.S. and 38% non-U.S.). Studies in HFSG intervention venues where foods were served were the minority of all included studies (18%), U.S. studies (32%), and non-U.S. studies (3%). A subset (9%) of U.S. studies assessed non-therapeutic patient meals in health care facilities.

### 3.2. Summary of U.S.-Based Studies

Descriptive characteristics, objectives, and descriptions of the U.S.-based HFSG interventions are provided in Table 2. Of the U.S. studies, nearly all took place in the continental U.S.—except one in Hawai’i [36] and one on U.S. and European military bases (this study was included only as a U.S. study) [40]. A handful of studies took a national or multi-location perspective on HFSG implementation [39,41,42,43], while the majority of studies focused on HFSG in various state, regional, or local populations. Some places were sites for multiple HFSG interventions: Los Angeles County in California [44,45] and New York City, New York [30,46,47]. Some studies were real-world evaluations of public policies (e.g., healthy beverage executive order) [45,48,49,50] or institutional programs (e.g., workplace sugar-sweetened beverage [SSB] ban, community sodium reduction programs) [29,30,36,38,39,41,43,46,51,52,53,54].

Key outcomes are summarized in Table 3a–e. Half of U.S. studies (50%) assessed multiple outcomes, with food environment being the most common (62%). Food environment outcomes were most frequently assessed using a pre-post study design with no control (62%), involving food environment audits to measure changes to food availability. Consumer behavior outcomes were measured in 29% of studies using indicators such as point-of-sales data to capture purchase quantity (e.g., volume), purchase quality (e.g., calories), or both; 80% of these used pre-post study designs with no control. Diet quality outcomes were most frequently assessed using pre-post designs with a control (50%). Health outcomes were least frequently assessed (12%), and 75% estimated health outcomes through simulation studies. Finally, financial implications related to total revenue and per-unit implementation costs were assessed in 38% of studies.

Below are summaries of study findings grouped by type of HFSG outcome evaluated and by type of primary HFSG intervention setting, as some studies took place in multiple settings but prioritized reporting of a primary setting. While we report specific findings from individual studies based on their primary setting(s), we also present overall summary proportions for each setting, resulting in a total sum that may exceed 100%.

#### 3.2.1. Findings from U.S. Studies Assessing Food Environment Outcomes

Food environment outcomes consider the availability of healthy food and beverage offerings regarding what is served or provided for sale at a venue. These (*n* = 21) were assessed in worksites (43%), health care facilities (43%), military settings (10%), community programs (48%), higher education (5%), recreation centers (5%), and child–youth programs (10%).

##### Worksites

Two studies used food environment audits to evaluate public policies that established nutrition standards for beverages and/or foods sold on government property [45,48]. Cradock et al. (2015) evaluated the Healthy Beverage Executive Order in Boston that eliminated all SSB sales and marketing in city-managed properties and services (e.g., worksites and community services) and restricted other beverages that were not unsweetened based on the Boston Public Health Commission’s traffic-light beverage classification system [48]. Wickramasekaran et al. evaluated the Los Angeles (LA) Healthy Vending Machine Nutrition Policy that set nutrition standards for all snacks and beverages sold in county-contracted machines [45]. Both studies found that availability of healthier products improved, but the LA policy did not reach full adherence by the end of the phase-in implementation [45,48].

Most studies used HFSG that were informed by established nutrition guidelines or frameworks for assessing healthfulness [36,43,44,64,65]. Lillehoj et al. used recommendations from the Institute of Medicine (IoM, now the National Academy of Medicine) to set healthy food availability standards, although the majority of vended items did not meet criteria across sites that included both worksites and community settings [65]. Jilcott Pitts et al. found that three years after implementation of Health and Sustainability Guidelines for Federal Concessions and Vending Operations from the U.S. Department of Health and Human Services and General Services Administration, all worksites successfully added healthier items, purchased more fresh produce, and modified recipes to meet guidelines [43]. However, success in federal worksites was dependent on having aligned values with clients, consumers, and vendors but was also constrained by staffing limitations and poor consumer feedback, resulting in more difficulty implementing changes. Elsewhere, two studies used variations of traffic light nutrition labeling systems based on the DGA to assess changes in healthy vending item inventory [36,64]. Donohoe, Mather, and McGurk found that inventory of the healthiest products increased during the time leading up to the launch of a healthy vending project [36]. Lessard et al. found that worksites met vending standards for healthful foods more quickly than for healthful beverages [64].

The only study to include a comparison group, Hopkins et al., conducted a process evaluation of a cluster RCT designed to establish organizational healthy food procurement processes—such as including HFSG language in subcontracts. Results showed that long-term intervention success for improving healthy snack availability was dependent on having support from both organizational leadership and middle management [44].

##### Health Care

Two studies examined HFSG in non-therapeutic patient meals [46,66], and three examined food procurement for vending machines, cafeterias, or cafes located in health care facilities [30,43,59]. Ranke et al. assessed the effects of the Balanced Menus Challenge, a meat purchasing reduction program designed to improve availability of sustainably produced protein; findings revealed that all participating hospitals reduced red meat on their menus primarily through one-to-one substitution [66]. Moran et al. (2015) evaluated the Healthy Hospital Food Initiative (HHFI) that used city government nutrition standards (based on federal guidance) to improve the nutritional quality of menus in New York City hospitals; they found that hospitals met key nutrition standards after HHFI implementation [46]. Moran et al. (2016) also conducted a process evaluation of HHFI to examine other access points (vending, cafeterias, and cafes) and found that increased availability of healthy food items, removal of unhealthy items, and reformulation to meet reduced sodium guidelines were most prevalent among private hospitals [30]. Jilcott Pitts et al. evaluated a similar intervention in hospitals across the U.S., finding that successful strategies were similar to those in federal worksites (summarized above); however, hospitals uniquely experienced support through intern staff capacity as well as resistance from consumers wanting comfort foods in hospital settings [43]. Cradock et al. (2022) also evaluated the impact of evidence-based nutrition guidelines in Massachusetts communities that emphasized sugar and sodium reduction strategies tailored to institutional capacity, finding overall improvements in low-sugar and low-sodium food and beverage availability in hospital cafeterias and vending machines [59].

##### Military

Two studies assessed food environment outcomes in military settings, both without comparison groups [40,55]. Belanger and Kwon evaluated outcomes of menu nutrition standards in a military dining facility using the Army’s “Go for Green” nutrition labeling system [55]. In the context of the Army’s Child and Youth Services program for Army families, Hanson et al. assessed a Healthy Menu Initiative for children and youth based on the DGA and other child nutrition guidelines using the Healthy Eating Index [40]. Overall, implementing HFSG improved the proportion of healthy items on food menus in both military settings [40,55].

##### Community Programs

The majority of studies in community settings assessed sodium reduction [29,38,41,54,57,62]. Jordan et al. evaluated the feasibility of implementing one of four predefined strategies that were part of a national sodium reduction program (Sodium Reduction in Communities Program [SRCP]), finding many sites (including congregate meal programs, worksites, and hospitals) during the program’s first two to three years began offering foods that were new, lower in sodium, or both [41]. Long et al. (2018) focused on SRCP-participating programs serving higher-risk populations; results showed that one year of reducing sodium in recipes accompanied declines in the overall mean sodium content of meals served per diner [54]. In a three-year follow-up study, Long et al. (2021) found that several sodium reduction strategies (e.g., standardizing food purchasing for low-sodium menu items) led to declines in mean sodium and mean energy served per diner [29]. Losby et al. evaluated sodium reduction strategies in food service and procurement for two counties in upstate New York for programs serving meals to older adults, finding that sodium served per meal declined over two years [38]. Brooks et al. assessed the proportion of higher-sodium prepackaged foods available—after a DGA-informed prepackaged sodium reduction intervention in vending machines and cafeterias in community institutions (including hospitals and recreation centers) with populations at higher risk for chronic disease, there was large variability in decreased high-sodium snack availability across different venues [57]. Hansotte et al. evaluated the effectiveness of recipe changes aligned with sodium reduction standards from the Family and Social Services Administration; the recipes used a different method of food preparation (speed-scratch cooking). Results revealed that meals in congregate meal programs and delivery programs in SRCP had reduced mean sodium content [62].

Durant et al. evaluated the impact of limiting sugary beverage availability in community settings in upstate New York counties, which included some hospitals and after-school programs; a difference-in-difference analysis found that soda availability declined more in intervention counties [61].

#### 3.2.2. Findings from U.S. Studies Assessing Consumer Behavior Outcomes

Consumer behavior outcomes measure quantity and quality changes in selecting or buying healthier and less healthy items following HFSG-related shifts in the food environment. These (*n* = 10) were assessed in worksites (60%), health care facilities (10%), military facilities (10%), community programs (40%), recreation centers (20%), and child–youth programs (10%).

##### Worksites

Berkowitz et al. found that after reducing entrée option sizes in urban Minnesota worksite cafeterias, selection of reduced-size entrées increased over the seven-week study [56]. Lessard et al. found that there were more purchases of healthful items in three public worksite vending machines in Delaware one year after implementing a healthy vending program based on DGA and IoM nutrition standards [64]. Wickramasekaran et al. found declines in average calories, sodium, and sugar in snack and beverage purchases after partial implementation of a 100% healthy vending policy in county-contracted vending machines in Los Angeles County [45]. Yan et al. evaluated a 100% healthy vending and micro-market model based on American Heart Association standards, finding declines in saturated fat, sodium, and sugar sold per snack [67].

##### Military

Belanger and Kwon et al. found that after partial implementation of military training menu standards (described above under food environment outcomes), soldiers more often chose the most nutritious options [55].

##### Community Programs

Jordan et al. found that sites (including congregate meal programs, worksites, and hospitals) implementing one of four strategies of a national sodium reduction program (SRCP) observed increases in purchases of low-sodium foods [41]. Karpyn et al. evaluated the introduction of healthy zoo concession stand items using Nutrition Environment Measures Survey criteria for fruits, vegetables, dairy, and packaged items. Findings showed that when animal cartoon characters were displayed, healthy items sold more frequently but remained less popular than unhealthy items [37]. Pharis et al. evaluated the Philadelphia Department of Public Health’s healthy vending standards that required vendors to offer a majority of items meeting DGA-based nutrition standards; results showed overall increases in healthy snack and beverage sales [49].

##### Recreation Centers

Volger et al. evaluated the cross-sectional difference in SSB volume purchased and consumed after the Barclays Center sporting arena in Brooklyn, New York, voluntarily adopted a 16 oz cap on SSB, finding that both measures were lower at Barclays compared to a control site sporting arena [47].

##### Child–Youth Programs

Laroche et al. evaluated the effects of a healthy concession intervention based on U.S. Department of Agriculture Team Nutrition standards for competitive foods during one year of high school athletics, finding gradual increases in healthier item purchases among attendees of high school football games [63].

#### 3.2.3. Findings from U.S. Studies Assessing Diet Quality Outcomes

Diet quality outcomes, such as reduced consumption of added sugars or increased whole grain intake, were assessed across six studies in worksites (33%), health care facilities (33%), military settings (50%), community programs (17%), higher education (17%), and child–youth programs (17%).

##### Worksites

Berkowitz et al. examined reducing entrée sizes in worksite cafeterias, finding that patrons had reduced intake of energy, saturated fat, and sodium [56]. Epel et al. evaluated a comprehensive workplace SSB ban—part of the University of California San Francisco’s Healthy Beverage Initiative—that eliminated sales in all campus and medical center venues, including hospital food services. Findings showed that SSB consumption declined overall even at long-term follow-up (10 months post-implementation) [53].

##### Military

Three studies assessed HFSG interventions in military dining facilities [55,58,60]. Belanger and Kwon et al. found that after partial implementation of healthy menu standards, soldiers consumed less sodium, vitamin C, and fewer calories both overall and from saturated fat [55]. Crombie et al. used a crossover RCT to evaluate an HFSG intervention that used DGA-informed standards to increase availability of healthful foods and standardized portions, finding that energy intake and saturated fat consumption were consistently lower for patrons at intervention facilities [60]. In a separate study in the same military dining facilities, Cole et al. assessed diet quality following implementation of DGA-informed performance-based menu standards designed to increase availability of nutrient-dense foods and reduce saturated fats, finding that patrons’ diet quality improved at the intervention facility compared to the control facility [58].

##### Community Programs

Durant et al. found no difference after one year in sugary beverage consumption attributable to an HFSG intervention that limited availability of SSB in vending machines at community settings in upstate New York counties [61].

#### 3.2.4. Findings from U.S. Studies Assessing Health Outcomes

Health outcomes, which include morbidity and anthropometric or biochemical indicators of diet-related chronic disease risk, were assessed across four studies in worksites (100%), health care facilities (75%), community programs (25%), higher education (50%), and child–youth programs (25%). All but one study used simulation models to estimate health outcomes [39,42,51].

##### Worksites

Epel et al., a pre-post study (with control) of a comprehensive workplace SSB sales ban at University of California San Francisco venues, found, after 10 months, an association with reduced average waist circumference among study participants but no improvements in body mass index (BMI) or insulin sensitivity [53]. Among simulation studies, Abrahams-Gessel et al. simulated health impacts of HFSG implementation at government worksite cafeterias. The researchers used meta-analyses of HFSG impact on dietary targets of fruit, vegetable, whole grain, processed meat, and SSB consumption, estimating that the federal workforce would have a lifetime reduction in cardiometabolic disease and related mortality [42]. Basu et al. simulated the 10-year impact on employee health from a workplace SSB sales ban in California health care organizations, also finding estimated lifetime reductions in diet-related diseases and mortality [51].

##### Community Programs

Yarnoff et al. estimated costs and cost-effectiveness of achieving predefined outcomes of a national sodium reduction program (SRCP) across all sites (including some workplaces, hospitals, and child–youth programs), finding that the longer SRCP is implemented, the greater are the projected reductions in cardiovascular disease and associated medical costs [39].

#### 3.2.5. Findings from U.S. Studies Assessing Financial Implications

Financial implications, such as changes to revenue, were assessed across 13 studies in worksites (69%), health care facilities (31%), military settings (8%), community programs (38%), higher education (8%), recreation centers (8%), and child–youth programs (15%). To estimate financial outcomes, 23% of studies used simulations.

##### Worksites

Abrahams-Gessel et al. simulated costs of HFSG implementation at government and large corporate worksite cafeterias, predicting that both five-year and lifetime horizons were cost-saving to employers [42]. Basu et al. simulated the impact of a workplace SSB sales ban, finding estimated cost savings due to averted health care and loss of productivity spending—provided workers purchase non-SSBs to offset revenue loss [51].

Of the real-world evaluations, nearly all focused on vending machine venues. Lessard et al. found a healthful food and beverage vending program had inconsistent monthly revenue gains and losses during the intervention [64]. Pharis et al. found that total revenues declined for both snacks and beverages over four years of implementing healthy vending standards based on DGA, which was part of a centralized city-wide vending contract in Philadelphia [49]. Wickramasekaran et al. found that revenue decreased in the two years following partial implementation of a healthy vending policy [45]. A healthy vending initiative based on American Heart Association standards, which targeted a worksite micro-market, was associated with increased mean monthly revenue [67]. Regarding federal worksite cafeteria venues, Jilcott Pitts et al. conducted qualitative interviews revealing more customer volume and profitability following a healthy vending initiative—despite price increases and upfront costs for training, labor, and equipment [43].

##### Health Care

Eneli et al. found that removing SSBs in all hospital-owned and contracted food service venues was associated with increased annual revenue from all beverages during the year post-implementation (excluding carbonated drinks) [52]. Jilcott Pitts et al. found similar results in hospitals compared to federal worksites, although HFSG had not been implemented as long (six months at hospitals compared to three years at worksites) [43].

##### Military

Cole et al. found that implementation costs per plate rose in the first six months of the intervention with DGA-informed performance-based nutrition standards; however, within one year the intervention was cost-saving [58].

##### Community Programs

Yarnoff et al. estimated the cost and cost-effectiveness of a national sodium reduction program’s (SRCP) predefined outcomes across all community partner sites (including some worksites, health care facilities, higher education settings, and child–youth programs). Findings showed that the program would be cost-saving if sustained for either up to 5 or 20 years after the end of the grant period [39]. Hansotte et al. found there were combined cost savings from two recipe modifications for sodium reduction over one year of implementation, which began at the end of the first year of SRCP [62]. Karpyn et al. found that increasing the availability of healthier packaged options at zoo concession stands had no impact on revenue [37].

##### Child–Youth Programs

Laroche et al. found that one year after a healthy concession intervention for high school sporting events—using nutrition guidelines for physical activity that introduced healthier food products and eliminated trans fats in some existing menu items—per-game sales of healthy items were higher than unhealthy items, although profit margins were lower [63].

### 3.3. Summary of Non-U.S. Studies

Descriptive characteristics, objectives, and descriptions of the HFSG interventions in non-U.S. studies are provided in Table 4. Of these non-U.S. studies, 21% were in North or South America, 50% in Europe or the Middle East, and 32% in Asia Pacific. In North America, excluding the U.S., all studies took place in Canada [35,68,69,70,71,72,73,74]. Some studies were real-world evaluations of public policies (e.g., Scottish Health Care Retail Standard) [32,75,76,77,78,79,80] or institutional programs (e.g., hospital ban on SSB) [31,68,69,81,82].

Key findings are summarized by outcome in Table 5a–e. Nearly half (44%) of non-U.S. studies assessed multiple outcomes, with food environment being the most common (53%). Food environment outcomes were most frequently assessed using a pre-post study design with no control (44%) and targeted vending machines (55%), cafeterias, canteens, and dining halls (50%), and retail outlets (50%). Consumer behavior outcomes were most often assessed with pre-post study designs with no control (38%) and RCT (38%); these studies measured changes in beverage and food purchasing behavior through sales data. Among the 29% of non-U.S. studies that assessed diet quality, 60% used pre-post study designs with control groups to measure changes in indicators like intake of salt, fruits, and vegetables. Only two studies, representing one unique intervention, assessed health outcomes [33,83]. Among the 29% of non-U.S. studies that assessed financial implications using revenue and sales data, 30% used RCT designs.

Below are detailed summaries of study findings, grouped by type of HFSG outcome evaluated and by type of primary HFSG intervention setting. While we report specific findings from individual studies based on their primary setting(s), we also present overall summary proportions for each setting, resulting in a total sum that may exceed 100%.

#### 3.3.1. Findings from Non-U.S. Studies Assessing Food Environment Outcomes

Food environment outcomes consider the availability of healthier and less healthy foods and beverages served or offered for sale at a venue. These studies (*n* = 18) assessed worksites (44%), health care facilities (33%), community programs (11%), and recreation centers (39%).

##### Worksites

All studies were RCTs [84,85,86]. Hollands et al. assessed a reduced portion size intervention in the United Kingdom (U.K.) that maintained the energy density of cafeteria items. The intervention was intended to apply to a range of menu items at worksites, but most sites attempted modifications for only main entrées [84]. In a study of a separate cluster of English worksites by the same investigators, Pechey et al. assessed an intervention aimed at reducing the energy density of cafeteria items without decreasing the number of items; findings showed that all sites targeted prepackaged and cold drinks for intervention but did not universally target other food categories [85]. In the Netherlands, Velema et al. found that 77% of eligible nudging strategies to introduce healthier products (including fruits, snacks, sandwiches, cheese, and meats) were implemented in intervention cafeterias [86].

##### Health Care

In Australia, Bell et al. evaluated a state policy based on school canteen guidelines for healthy vending and retail offerings, designed to improve food environments in health care facilities; findings revealed that neither type of venue (vending, retail outlets) met standards after implementation [75]. Another healthy vending policy evaluated by Boelsen-Robinson et al. (2017) found that the variety of healthy foods and beverages was less than the variety of unhealthier products, despite restrictions on how many unhealthy products could be offered [76]. Cranney et al. (2020) evaluated another Australian state policy that promoted SSB removal from health facilities, finding that after the policy went into effect there was a statistically significant increase in the number of hospital retail outlets that removed SSB from inventory [77]. Miller et al. also evaluated a state policy that limited the supply and promotion of energy-dense, nutrient-poor foods to 20% of all displayed products in food supply areas. Findings showed that, compared to larger facilities, smaller health care facilities were more likely to fully implement the policy and increase availability of healthy foods and drinks [78]. In Canada, Dojeiji et al. evaluated a regional program for healthier hospital food services to improve cardiovascular population health; findings showed that the variety of healthier products was limited despite most sites’ compliance with the program’s “bronze” standards for food procurement, preparation, and availability [69]. Stead et al. evaluated a retail policy based on Scottish government nutrition criteria to promote healthier food and drink product supply, finding that most shops became compliant and sourced healthier product alternatives from smaller suppliers [79].

##### Community Programs

Katz-Shufan et al. (2022) evaluated a nutrition environmental intervention that modified recipes served in a communal dining room according to Israeli Ministry of Health dietary recommendations, finding that modifications led to increased fruit and vegetable portions and reduced sodium, saturated fat, and energy in meals [34]. In South Korea, Seo et al. evaluated a sodium reduction program for a congregate meal service center for elderly patrons, finding that new menus (still serving culturally appropriate meals) excluding processed foods and ingredients had lower sodium per soup serving [87].

##### Recreation Centers

Studies in Australia and Canada assessed outcomes in recreational sites. In Australia, Boelsen-Robinson et al. (2021) evaluated outcomes of SSB reduction using state government traffic-light-style nutrition guidelines for beverages; data showed that one-third of sites removed all SSB, 87% removed all soft drinks, and, on average, sites offered 4.4 fewer red drink varieties for sale [31]. Wolfenden et al. used an RCT design to examine a healthy canteen strategy at community sporting clubs, finding that intervention clubs improved availability of fruit and vegetable products [88].

In Canada, McIsaac et al. evaluated the voluntary adoption of Healthy Eating in Recreation and Sport Settings (HERSS) using nutrition guidelines for public schools, finding that food environments either worsened or remained unchanged [70]. Olstad et al. (2015) examined how an urban community recreation facility applied provincial nutrition guidelines to concession stand offerings, finding that healthy offerings increased [71]. Separately, Olstad et al. (2019) used an RCT design to evaluate how capacity-building enhanced adoption of nutrition guidelines for children and youth in Alberta province, finding that all facilities that developed nutrition policies were among those that received a capacity-building intervention [72]. In a study with a subset of these facilities, Lane et al. found that the facilities with healthy vending contracts that aligned with provincial guidelines had the greatest reductions in availability of unhealthy products [35]. Naylor et al. also assessed a capacity-building intervention within British Columbia communities that received grants and implementation support to provide health-promoting environments in public buildings. Communities that received resources, training, and technical support were more successful at adopting healthy policies and increasing healthy vending product availability than communities not receiving assistance [73].

#### 3.3.2. Findings from Non-U.S. Studies Assessing Consumer Behavior Outcomes

Consumer behavior outcomes measure changes in selection of healthier and less healthy items following HFSG-related shifts in the food environment. These studies (*n* = 13) assessed worksites (69%), health care facilities (31%), higher education (15%), and recreation centers (23%).

##### Worksites

In England, Hollands et al. found no improvements in daily calories purchased after a portion size reduction RCT that maintained the energy density of items offered at worksite cafeterias [84]. In comparison, Pechey et al. conducted an RCT that reduced the energy density of cafeteria items in a separate cluster of worksites, finding that total daily energy purchased decreased at follow-up [85]. Reynolds et al. evaluated an availability and portion size intervention for workers in U.K. supermarket distribution centers in a stepped wedge RCT, finding that energy purchased decreased the most when higher-energy products were both replaced with lower-energy alternatives and reduced in size [89]. Velema et al. also measured the effect of increasing availability and visibility of healthier sandwiches, salads, cheese, and meats in Dutch worksite cafeterias, finding that three of seven categories of healthier options were purchased more frequently [86].

##### Health Care

In Australia, Boelsen-Robinson et al. (2017) evaluated a healthy hospital vending policy based on Victoria state government dietary guidelines requiring at least half of displayed items to be healthy, finding that purchases of least healthy items declined [76]. Tinney et al. evaluated a health service’s ban on packaged SSB, also based on Victoria state guidelines, finding that while healthier beverages were purchased more often, many customers compensated by purchasing SSB elsewhere [80]. Cranney et al. (2021) found that after implementation of a statewide healthy food and beverage policy using New Zealand national dietary guidelines that removed SSB in cafeterias, vending machines, and other outlets, there were small but insignificant improvements to purchasing healthy items [32]. In the U.K., Simpson et al. evaluated a multi-component intervention to increase healthy food availability in one hospital retail outlet using national guidelines, finding that sales increased for healthy food options and decreased specifically among sweets and chocolate products [90].

##### Higher Education

Di Sebastiano et al. evaluated a healthy beverage initiative at an urban university campus in Canada that removed SSB without naturally occurring nutrients in one dining hall, finding that students purchased more healthier beverages without other compensatory purchasing behaviors [68]. Vermote et al. evaluated portion size reduction for French fries offered at a university dining hall in Belgium, finding no significant difference in French fry purchasing during the intervention compared to baseline [91].

##### Recreation Centers

In Australia, Boelsen-Robinson et al. (2020) found that an SSB reduction initiative—using institutional nutrition guidelines for high-calorie carbonated beverages, flavored milk, and less than 99% juice beverages—led to long-term declines in sales of SSB and sugar from packaged drinks; however, declines in sugar content of purchases attenuated after a year [82]. In another Australian state, Wolfenden et al. used intent-to-treat analyses to determine that patrons bought more healthy foods and non-sugar-sweetened beverages at sporting clubs with a healthy canteen strategy promoting availability of fruits, vegetables, and non-sugar-sweetened drinks [88]. In Canada, Olstad et al. (2015) found that after an urban community recreation facility applied Alberta province nutrition guidelines to concession stand offerings, sales of healthy items increased [71].

#### 3.3.3. Findings from Non-U.S. Studies Assessing Diet Quality Outcomes

Diet quality outcomes, such as reduced consumption of added sugars, were assessed across 10 studies in worksites (60%), health care facilities (10%), military settings (20%), community programs (20%), and higher education (10%).

##### Worksites

Beer-Borst et al. evaluated a joint educational–environmental nonrandomized trial to apply national Swiss guidelines for sodium reduction in communal catering settings; findings showed that small changes in salt intake were not statistically significant [92]. Geaney et al. (2016) evaluated an environmental dietary modification to improve healthy offerings and portion sizes for Irish manufacturing worksites, finding that employees at intervention sites had significant reductions in salt and saturated fat intakes [83]. In Denmark, Lassen et al. (2014) found that workers eating lunch at a worksite canteen, which became certified to use a national label for healthy food, had overall improvements in dietary quality based on fruits, vegetables, sodium, and energy density compared to an uncertified worksite canteen [81]. In Italy, Vitale et al. examined the long-term impact of an intervention that both modified canteen recipes per the Mediterranean diet and trained food service staff in healthy food preparation; findings revealed that there was sustained improvement in consuming healthier, less-processed foods [93].

##### Health Care

In Canada, Vanderlee et al. compared the dietary quality of patrons at intervention hospital cafeterias that changed recipes and removed deep fryers to cafeterias that did not. Findings from cross-sectional exit surveys showed patrons at intervention sites consumed significantly less energy, sodium, and saturated fat [74].

##### Military

Both northern European studies used a pre-post design with a control group. Bingham et al. evaluated an intervention for military dining halls in Finland that increased the supply of healthy foods, finding that conscripts eating at intervention sites had improved consumption of fresh vegetables and fiber-rich grains [94]. In Norway, Uglem et al. evaluated a kitchen environment intervention to increase availability of healthy food items and support added training to prepare vegetables in appealing ways, finding that military recruits at intervention sites had higher intake of vegetables, fruits, and whole grains [95].

##### Community Programs

Katz-Shufan et al. (2020) found that recipe changes led to improved lunch nutritional quality scores and more consumption of fruits and vegetables for kibbutzim residents dining in intervention cafeterias [96]. In Seoul, Seo et al. found that elderly patrons of a congregate meal service center—which modified recipes as part of the sodium reduction program of culturally relevant foods—consumed less sodium-heavy foods [87].

##### Higher Education

Vermote et al. evaluated a portion size reduction intervention on French fries offered at a university dining hall in Belgium, finding that there was significantly less French fry consumption [91].

#### 3.3.4. Findings from Non-U.S. Studies Assessing Health Outcomes

Health outcomes were assessed only in one intervention, Food Choice at Work (FCW), which resulted in two publications [33,83]. Geaney et al. first found that the combination of both nutrition education and environmental dietary modifications (including menu reformulation, discounts on healthy options, and portion sizing) led to small but significant reductions in BMI [83]. Fitzgerald et al. assessed the cost-effectiveness of FCW, finding that the combined intervention led to estimated improvements in quality-adjusted life years, but improvements were greatest through environmental dietary modifications [33].

#### 3.3.5. Findings from Non-U.S. Studies Assessing Financial Implications

Financial implications, such as revenue from beverages and food, were assessed across 10 studies in worksites (50%), health care facilities (20%), community programs (10%), higher education (10%), and recreation centers (30%).

##### Worksites

Fitzgerald et al. assessed the cost-effectiveness of FCW in Irish manufacturing worksites, finding that adding intervention components cost employers more. However, the greatest reductions in absenteeism were observed when workers received both environmental dietary modification and nutrition education [33]. Reynolds et al. found small declines in revenue—depending on the number of intervention components implemented in an RCT of healthy food availability and portion size reductions—in U.K. supermarket distribution worksites [89]. Pechey et al. found no evidence of revenue impact in an RCT of increasing availability of healthier, lower-energy food options in English worksite cafeterias [85].

##### Health Care

In Australia, Boelsen-Robinson et al. (2017) found a healthy vending policy using state government dietary guidelines had no impact on revenue due to a renegotiation on commission percentages for vended items [76]. In the U.K., Simpson et al. found that following a healthy food availability intervention, based on national guidelines in one hospital retail outlet, total sales increased up to 27% during the 10-month post-intervention period [90].

##### Community Programs

Katz-Shufan et al. (2022) conducted a follow-up study to their initial food environment and dietary assessment in a kibbutzim communal dining hall [96], finding that there was a small incremental increase in cost per serving associated with the recipe modifications [34].

##### Higher Education

Di Sebastiano et al. evaluated a healthy beverage initiative at an urban university campus dining hall that eliminated SSB without naturally occurring nutrients; findings showed that purchasing declines corresponded with declines in total revenue [68].

##### Recreation Centers

Olstad et al. (2015) found no evidence of lost revenue after an urban community recreation facility applied Canadian provincial nutrition guidelines to concession stand offerings [71]. In New South Wales, Australia, Wolfenden et al. also found no effect of a healthy canteen strategy on annual club revenue [88]. Boelsen-Robinson et al. (2020) found that an SSB reduction initiative using institutional nutrition guidelines in Victoria, Australia, led to long-term declines in sales of all drinks [82].

**Table 4:** Descriptive information for included studies conducted outside the United States (N = 34) representing 29 unique interventions.

Citation	Author (Year)	Study Objective	Location	Study Design	Intervention Description
[92]	Beer-Borst et al. (2019)	Conduct an impact evaluation of an educational–environmental workplace sodium reduction trial at public and private workplaces with catering facilities.	German-speaking Switzerland	Pre-post, control (1 yr)	The “Healthful & Tasty: Sure!” program provided coaching for catering teams to apply national guidelines for salt reduction in communal catering settings.
[75]	Bell et al. (2013)	Describe the impact of intervention to improve availability and labeling of healthier food and beverage items in health care facilities’ vending machines and food outlets.	Hunter New England local health district, New South Wales, Australia	Pre-post, no control (vending: 4 yrs; outlets: 2 yrs)	New South Wales (NSW) policy required public health sites to provide healthier options and restrict unhealthy options from vending machines and retail outlets. Must offer ≥ 80% “green” or “amber” drinks and ready-to-eat food items, while restricting serving sizes of “red” drinks to ≤375 mL. Healthier options must also be labeled. Standards adapted from school canteen guidelines.
[94]	Bingham et al. (2012)	Assess effects of intervention on conscripts’ eating habits in military dining halls.	Finland	Pre-post, historical control (follow-up at 8 wks and 6 mo)	Targeted supply of healthy foods in main sources of food in the military. Specific nutrition goals: increase fruit and vegetable consumption, increase fiber intake, and decrease saturated fat and sugar intake.
[76]	Boelsen-Robinson et al. (2017)	Examine the change in purchasing of healthy and unhealthy foods and beverages from hospital vending machines after the healthy vending machine policy.	Victoria, Australia	Interrupted time series (30 mo prior, 12 mo post)	Victorian public hospitals follow Healthy Choices (food and drink guidelines). Must reduce the proportion of unhealthy items for sale and increase the availability of healthy items. Guidelines classify items based on nutrient quality into “red” (limit, ≤20%), “amber” (choose carefully), and “green” (best, ≥50%).
[31,82]	Boelsen-Robinson et al. (2020); Boelsen-Robinson et al. (2021) ^a^	Assess the impact of SSB reduction initiatives on customer beverage purchasing patterns in YMCA aquatic and recreation centers.	Victoria, Australia	Interrupted time series (2 yr prior, 1 yr initiative, 1 yr post)	Adapted from state government guidelines, the initiative focused on reducing the availability of “red” packaged SSB: non-diet carbonated beverages, flavored water and milk, and fruit drinks (<99% juice or >250 mL). Reduced sports drinks (<10%) and increased “green” options (>70%).
		Assess the extent of implementation and customer acceptability of the SSB reduction initiative.		Pre-post, no control (6 mo)	The SSB reduction initiative was promoted as a “soft-drink-free summer” campaign. Introduced and maintained new drink options and fridge displays.
[32,77]	Cranney et al. (2020); Cranney et al. (2021) ^a^	Evaluate the change in SSB availability after the SSB removal mandate, part of the statewide policy Healthy Food and Drink in New South Wales Health Facilities for Staff and Visitors Framework.	New South Wales, Australia	Pre-post, no control (audit: 2 mo, consumer surveys: 3–4 mo)	The framework aimed to increase availability/promotion of healthy items in health facility food outlets. Phase 1 of implementation: remove prepackaged SSB.
		Examine changes in food purchasing behaviors after policy introduction.		Pre-post, no control Baseline (5–8 mo prior), follow-up (4–6 mo after)	Phase 2 of implementation: implement all 53 healthy food and drink practices. The ultimate goal is to increase the availability of “everyday” (healthy) and reduce “occasional” (unhealthy) options.
[68]	Di Sebastiano et al. (2020)	Examine revenue impact and potential compensatory purchasing behavior following the Healthy Beverage Initiative at an urban university campus.	Vancouver, British Columbia, Canada	Pre-post, control (baseline: two semesters; follow-up: two semesters)	Removed “red” (SSBs without naturally occurring ingredients) from the residence dining hall and replaced with “green”/“yellow” beverages. Yellow: non/lightly sweetened or with naturally occurring nutrients. Green: water, coffee/tea, plain milk, and milk alternatives.
[69]	Dojeiji et al. (2017)	Describe implementation strategies, successes, challenges, and limitations of the Healthy Foods in Champlain Hospitals Program.	Champlain region of Eastern Ontario, Canada	Pre-post, no control (15 mo)	Nutrition standards are phased in over time via bronze, silver, and gold benchmarks. Key areas of focus include providing calorie/sodium information; increasing availability of whole grains, vegetables, and fruit; removing deep fryers and all fried foods; reducing sugar and sodium; and reducing high-calorie beverages.
[33,83]	Geaney et al. (2016); Fitzgerald et al. (2018) ^b^	Assess the comparative effectiveness of system-level dietary modification and nutrition education through the Food Choice at Work (FCW) intervention at the manufacturing worksite.	Cork, Ireland	Pre-post, control (7–9 mo)	Environmental dietary modification included five elements: menu modification by limiting saturated fat, sugar, and salt; increases in fiber, fruit, and vegetable quantity; price discounts for whole fresh fruit; strategic positioning of healthier alternatives; and portion size control.
		Assess the cost-effectiveness of FCW.		Pre-post, control (9 mo)	
[84]	Hollands et al. (2018)	Assess feasibility, acceptability, and impact on energy purchased of portion size reduction in the food and grocery industry’s worksite cafeteria at office and manufacturing sites.	England, United Kingdom	Stepped-wedge RCT (4-week baseline, 3–13 wk intervention based on staggered 2-week periods)	Reduce ≥10% portion sizes without changing the energy density of foods available in cafeterias from targeted categories: main meals, sides, desserts, and cakes. Keep a range of food products. Use proportional pricing to maintain value for money for smaller portions.
[34,96]	Katz-Shufan et al. (2020); Katz-Shufan et al. (2022) ^b^	Impact of Nutrition Environmental Kibbutzim Study (NEKST) intervention on diet quality and diversity in communal dining rooms.	Israel	Pre-post, control (3 mo)	Recipes were modified according to dietary recommendations, mainly by reducing sodium, sugar, and saturated fat. Other components included environmental changes in the dining room (location of dishes, healthy labeling with a green “like”), health communications, and an education program for both control/comparison dining rooms.
		Evaluate the impact of NEKST on the nutritional content of dishes available and the incremental costs of recipe modifications.		Pre-post, control (3 mo)	Modifications included lower-fat meat and dairy products, fiber-rich substitutions, adding vegetables, and reducing sodium- and sugar-rich cooking ingredients and sauces.
[81]	Lassen et al. (2014)	Evaluate the impact of the Danish Keyhole certification program in the hospital worksite canteen on the nutritional quality of lunch meals.	Denmark	Pre-post, control (6-week, 6-month follow-up)	The Danish Veterinary and Food Administration introduced labels (Nordic Keyhole) for freshly prepared healthy meals. Foods eligible for labeling must fulfill certain conditions: maximum amounts of fat, salt, and sugars + minimum amounts of dietary fiber and whole grains in 25 different food groups. To use the label, canteens must go through certification, employees must also be certified, and at least one labeled menu must be on the daily menu.
[70]	McIsaac et al. (2018)	Assess the impact of voluntary nutrition guidelines in recreational and sport settings on access to healthy foods and beverages.	Nova Scotia, Canada	Pre-post, no control (1 yr)	Healthy Eating in Recreation and Sport Settings, based on guidelines for Nova Scotia Public Schools, classified all foods and beverages in vending machines and concessions as Do Not Sell (deep-fried foods, energy drinks, and relaxation beverages), Minimum (<10% stock; high added fat, sugar, and sodium), Moderate (≤40% stock; some processing, contains essential nutrients), and Maximum (≥50% stock; high in essential nutrients, low in saturated/trans fats, minimally processed, little/no added fat, sugar, and sodium).
[78]	Miller et al. (2015)	Evaluate the implementation of statewide policy (A Better Choice) in increasing the availability of healthy foods and drinks in public health care facilities.	Queensland, Australia	Cross-sectional	The goal was to increase supply/promotion of healthy foods and drinks and limit supply/promotion of energy-dense, nutrient-poor foods in all food supply areas. Nutrient profiling based on energy, saturated fat, sodium, and fiber per serving is used to categorize foods as “green” (best), “amber” (choose carefully), and “red” (limit). the policy limited red options (≤20%) and only permitted promotions for green options.
[73]	Naylor et al. (2015)	Assess the impact of capacity-building intervention (Healthy Food and Beverage Sales [HFBS]) in recreation and sport facilities on food environment and food policy development.	British Columbia, Canada	Pre-post, control (8 mo)	HFBS supported implementation of British Columbia’s voluntary guidelines for food sales in public buildings. At least half of available vending products should be from the healthiest “Choose Most” category, up to half from “Choose Sometimes,” and none from the “Choose Least” and “Not Recommended” categories. HFBS communities received a USD 7500 grant and implementation support (framework, training, resources, and technical assistance).
[71]	Olstad et al. (2015)	Assess change in sales of healthy foods after increasing availability in a community recreation facility in an urban setting.	Alberta, Canada	Pre-post, no control (40 days)	Alberta’s Nutrition Guidelines for Children and Youth classified foods and beverages as “choose most often,” “choose sometimes,” and “choose least often” based on energy, fat, saturated fat, trans fat, fiber, protein, sodium, calcium, vitamin D, and artificial sweeteners. Availability of healthy items was 9.1% during the pre- and post-intervention periods and 25% during the intervention period.
[35,72]	Olstad et al. (2019); Lane et al. (2019) ^c^	Test the impact of capacity-building intervention in enhancing the implementation of provincial nutrition guidelines in public recreation and sport facilities. Assess whether guidelines were associated with positive changes.	British Columbia, Nova Scotia, and Alberta, Canada (intervention); Ontario, Canada (control)	RCT embedded in natural experiment (18 mo)	All three provincial guidelines classified the healthfulness of foods with nutrient profiling schemes and provided guidance for increasing the availability, accessibility, and promotion of healthier foods. Measured food environment quality by classifying products as Sell Most (nutrient-rich and lower in sodium, sugar, and fat), Sell Sometimes (contains essential nutrients and higher in sodium, sugar, and fat), and Do Not Sell (energy-dense and nutrient-poor and high in sodium, sugar, and fat) in vending and concessions; also used the Nutrition Environment Measures Survey-Restaurant reduced item audit in concessions.
		Sub-study: measure influence of healthy vending contracts on nutrition quality of products sold.		Pre-post, control (18 mo)	
[85]	Pechey et al. (2019)	Assess feasibility and acceptability of increasing the proportion of healthier, lower-energy options for energy purchased at worksite cafeterias.	England, United Kingdom	Stepped-wedge RCT (4-week baseline, 3–13-week intervention based on staggered 2-week periods)	Aimed to keep the total number of options constant while improving the availability of healthier cooked meals (300–500 kcal; limit to one less healthy meal and side per day); sandwiches (<350 kcal, 50% of options); snacks (120–150 kcal, 50% of options); and cold drinks (<50 kcal, 50% of options).
[89]	Reynolds et al. (2021)	Test the effectiveness of availability and portion size interventions on the energy density of food and drinks purchased at worksite cafeterias in supermarket distribution centers.	United Kingdom	Stepped-wedge RCT (25 wk: minimum 4-week baseline, 8-week availability intervention, 4–13-week availability + size intervention)	Availability: replaced higher energy with lower energy products to change relative availability among main meals, side dishes, cold drinks, sweet/savory snacks, desserts, and bakery items. Portion size: reduced size of higher energy products (≥10%) for main meals, sides, desserts, and bakery items.
[87]	Seo et al. (2016)	Evaluate intervention to improve eating habits among the elderly by reducing sodium intake and providing nutrition education at congregate meal service center.	Seoul, South Korea	Pre-post, no control (4 wk)	Modified lunch menus based on healthy eating and reduced salt intake. New menus excluded processed foods and ingredients (salted dry fish, pickled vegetables [kimchi]) and incrementally reduced the sodium content of soup/stew weekly.
[90]	Simpson et al. (2018)	Measure the impact of increasing healthy food products through multi-component intervention on food and drink purchasing in hospital retail outlets.	London, United Kingdom	Longitudinal (pre-intervention: 2 mo; early post-intervention: 2 mo; late post-intervention: 10 mo)	Introduced healthier products, limited portion size of unhealthy options, reduced promotion of unhealthy options, incentivized healthier choices in meal deals, and increased prominence of healthier options. Guidelines for “healthy” based on government guidance: products must contain less than 20 g fat, 5 g saturated fat, 5 g added sugar, and 1.5 g salt per 100 g. Other components of intervention included limiting portion size of unhealthy options, reducing promotion of unhealthy options, including healthy options in meal deals, and improving placement of healthy options.
[79]	Stead et al. (2020)	Evaluate the impact and implementation of the Healthcare Retail Standard (HRS) on the food and drink product range.	Scotland, United Kingdom	Early-post, no control (18-month implementation period)	HRS required ≥50% food items and ≥70% drinks (excluding water) to meet Scottish government nutrition criteria. Only food items meeting nutrition criteria can be promoted.
[80]	Tinney et al. (2022)	Assess the impact of the SSB ban on the availability and purchasing of packaged beverages and self-reported SSB consumption.	Victoria, Australia	Pre-post, no control (drink sales: 6-month pre vs. 12-mnth post; consumption: 6-month post)	Mandatory removal of SSB according to Victoria’s Healthy Choice Guidelines. SSB is defined as carbonated soft drinks, flavored water, nutrient water, iced tea, and sports and energy drinks, all with added sucrose. Larger-sized milk-based and alternative drinks with added sugar and larger-sized 99% juice without added sugar were exempt.
[95]	Uglem et al. (2014)	Measure the impact of the healthy food availability intervention on military recruits’ food intake at the military canteen.	Norway	Pre-post, control (5-month intervention for two consecutive enrollments)	Increased availability of healthy food items through a self-service salad bar at lunch; new main/side dishes with vegetables at dinner; and whole-grain, higher-fiber bread at all meals. Kitchen staff received training to prepare new dishes and present additional vegetables in appealing ways.
[74]	Vanderlee et al. (2014)	Assess the impact of nutrition displays for energy, sodium, saturated and total fat at point-of-sale in hospital cafeterias on food purchasing and consumption.	Ottawa, Ontario, Canada	Cross-sectional (8-month post-intervention)	The intervention cafeteria reformulated some food recipes and removed the deep fryer from the kitchen to increase the availability of healthier food items. A health logo marked items that met nutritional standards, and a prominent digital menu board displayed all nutrition information for all items. The control cafeteria had a paper menu labeling a limited selection of items.
[86]	Velema et al. (2018)	Assess the effect of nudging strategies, including product-specific changes, in “Worksite Cafeteria 2.0” on purchasing behavior at cafeterias in companies with contracted external catering.	Netherlands	RCT (3-week baseline, 12-week intervention)	To improve products: visibly offer ≥ 1 “better choice” product; also offer smaller portions of warm lunch meals, fruits and vegetables, free water, ≥60% visible share of healthy “better choice” products, and warm (not packaged) snacks ≤3 days/week.
[91]	Vermote et al. (2018)	Investigate the effect of portion size reduction on French fry consumption and plate waste, satiety, and caloric intake at a university dining hall.	Brussels, Belgium	Pre-post, no control (4 days each for baseline and intervention)	Reduced French fries portion by 40 g (20%) using smaller volume bags.
[93]	Vitale et al. (2018)	Evaluate the effectiveness and long-term (3-year) impact of a healthy food choices intervention based on the traditional Mediterranean diet at the food company’s worksite canteen.	Pedrignano, Parma, Italy	Pre-post, no control (6 mo, 3 yr)	To increase availability and promotion of healthy food choices: provided training for canteen staff for healthy food preparation and nutrition guidelines; improved dietary quality of existing recipes; included new dishes based on the traditional Mediterranean diet; and used logos identifying new healthy dishes on menus.
[88]	Wolfenden et al. (2015)	Measure the effect of a healthy canteen strategy for the availability and purchases of fruits and vegetables and healthy beverages at the community recreation center for football clubs.	New South Wales, Australia	RCT, stratified by club type and region (2.5 sporting seasons)	Intervention clubs provided six fruit- and vegetable-based options and non-SSB for sale. Required that ≥75% of non-alcoholic drinks in canteen fridges were not SSB and were positioned in the top half of the fridge. Recommendations included substituting higher fat/energy products with lower fat/energy options, using competitive pricing for healthy options, and using fact sheets to normalize healthy purchasing.

HFSG: healthy food service guidelines; RCT: randomized controlled trial; SSB: sugar-sweetened beverage. ^a^ These separate publications evaluated the same HFSG program or intervention to reduce SSB consumption, so they are presented together as one pair of studies of one unique intervention. Both of the first studies published on HFSG were impact evaluations of the SSB reduction programs. The second studies were follow-up evaluations that focused on evaluating customer experiences with HFSG. ^b^ These pairs of publications evaluated the same HFSG program, so they are presented together as representing one unique intervention. ^c^ Lane et al. (2019) [35] was a sub-study that used data from the HFSG evaluation conducted in Olstad et al. (2019) [72], so the two publications are presented together as representing one unique intervention.

**Table 5:** Key findings from included studies conducted outside of the U.S.

(a). Key Findings from Included Studies Conducted Outside of the U.S. on Food Environment Outcomes of HFSG Interventions (N = 18)								
Author (Year)	HFSG Setting(s) ^a^							Key Findings: Food Environment
	WS	HC	M	CP	HE	Rec	Ch/Y	
Bell et al. (2013) [75]		X						Most vending machines did not meet the health policy standard of 80% amber/green (healthier/healthiest) food or drinks or labeling, but the proportion of amber/green drinks significantly improved. Outlets did not have significant improvements in the ratio of amber/green drinks and foods. The majority of outlets did not meet the 80% standard at follow-up.
Boelsen-Robinson et al. (2017) [76]	X	X						After implementing a healthy vending policy that mandated ≥50% green (healthiest) products and ≤20% red (need to limit) products, the variety of available green items was lower than for red items.
Boelsen-Robinson et al. (2021) [31]						X		10 of 30 centers removed all SSBs; 26 removed all soft drinks. Red (least optimal) drink varieties were reduced by 4.4 (*p* < 0.05) across all centers; green (optimal) drink availability increased by 1.4 (*p* < 0.05). Among centers that did not meet SSB reduction goals, they reduced availability of red drinks (−3.8). Half of all centers did not change, or they decreased the variety of green drinks.
Cranney et al. (2020) [77]	X	X						The proportion of outlets that removed SSB increased from 58% to 96.3% (*p* < 0.001).
Dojeiji et al. (2017) [69]	X	X						In this study, 21 of the 23 sites have met the bronze benchmark. Regarding vending machines: at halfway, only 35% and 15% of the assessed vending machines met requirements for reduced portion size of high-calorie beverages and reduced proportion of sweets and snacks, respectively. Although 96% became compliant after another 7 months, the availability of diverse, healthier products was limited.
Hollands et al. (2018) [84]	X							Portion size reductions were difficult to implement across the entire range of available products (prepackaged products are in unmodifiable units, and there is an additional burden for staff on menu changes and serving portions). Main meals were the only targeted category where all sites attempted implementation.
Katz-Shufan et al. (2022) [34]				X				Recipe changes led to reduced sodium in 14 recipes, saturated fat in 11, and energy in 14.
McIsaac et al. (2018) [70]						X		Food and beverage environments worsened post-guideline. The majority of vending foods/beverages were of minimum nutrition at baseline and follow-up, and Do Not Sell (DNS) vending beverage stock increased. The majority of concession products were minimum and DNS items. the proportion of DNS and moderate foods in concessions increased significantly at follow-up. The proportion of maximum nutrition concession beverages decreased at follow-up.
Miller et al. (2015) [78]	X	X						Of the managers that responded, 25% reported full implementation of A Better Choice in all food supply areas, 78% reported majority implementation, and 20% reported up to half implementation. Managers of small facilities were more likely to fully implement the policy than large facilities.
Naylor et al. (2015) [73]						X		Healthy Food and Beverage Sales (HFBS) communities had significantly greater capacity for implementing guidelines than comparison groups. HFBS communities adopted more healthy food policies (10% to 48%), increased healthy vending products (11% to 15%), and decreased unhealthy products (56% to 46%).
Olstad et al. (2015) [71]						X		Availability of healthy items increased from 9.1% to 25.0% and 44.4% within the target concession.
Olstad et al. (2019) [72]; Lane et al. (2019) [35] ^b^						X		In this study, 17.6% of guideline + capacity-building intervention (GL + CBI) facilities codified new nutrition policies, none among guideline only (GL-ONLY) or comparison (NO-GL) facilities. No changes for concession venues. In vending machines of GL + CBI facilities, the proportion of DNS snacks declined, and the proportion of Sell Sometimes snacks increased relative to GL-ONLY and NO-GL facilities. No facilities significantly improved the proportion of Sell Most snacks, although GL + CBI had minor increases.
						X		Facilities with consistent healthy vending contracts (HVCs) or that had adopted HVCs had significantly greater decreases in DNS product availability.
Pechey et al. (2019) [85]	X							All sites intervened (increased the proportion of lower-calorie options) on prepackaged and cold drinks, five sites intervened on snacks, and one site intervened on sandwiches.
Seo et al. (2016) [87]				X				Modified menus had −166 mg sodium per soup serving.
Stead et al. (2020) [79]	X	X						All but one shop became Healthy Retail Standard compliant by the end of the implementation period. The number of chocolate products declined between early implementation and 4 months post-implementation, but there were no increases in fruit products. Healthier product alternatives boosted business for smaller suppliers of niche products. Centralized processes were named as inflexible for not allowing outlet managers to implement guidelines in different settings.
Velema et al. (2018) [86]	X							In this study, 77% of all nudging strategies (including product, place, price, and promotion) were implemented in intervention cafeterias.
Wolfenden et al. (2015) [88]						X		Based on intent-to-treat analyses, intervention canteens had significant increases in availability of fruit and vegetable products. No significant differences in availability of non-SSBs.
**(b) Key findings from included studies conducted outside of the U.S. on consumer behavior outcomes of HFSG interventions (N = 13)**								
**Author (Year)**	**HFSG Setting(s) ^a^**							**Key Findings: Consumer Behavior**
	**WS**	**HC**	**M**	**CP**	**HE**	**Rec**	**Ch/Y**	
Boelsen-Robinson et al. (2017) [76]	X	X						Overall, there was a 55.7% reduction in red item sales. Based on pre-policy trends, 56% fewer red drinks, 21.9% more green drinks, and 21.9% fewer amber drinks were sold after implementation. Reduction in red drinks: −846.9 L volume, −100.7 kg sugar sold per month.
Boelsen-Robinson et al. (2020) [82]						X		At the end of the intervention, significant improvements were seen: −46% volume sales of red drinks and −35% sugar purchased through packaged drinks. Reduction in sugar purchases attenuated after 1-year post-implementation.
Cranney et al. (2021) [32]	X	X						There was a non-significant increase in the proportion of “everyday” food purchases and non-significant adjusted odds of purchasing these items for visitors vs. staff (−22%), young vs. older adults (−29%), non-tertiary educated (−29%), and non-English speakers at home (+52%).
Di Sebastiano et al. (2020) [68]					X			First semester post-Healthy Beverage Initiative (HBI): increased green beverage sales. Second semester post-HBI: increased yellow beverage sales. Limited evidence of compensatory SSB purchasing.
Hollands et al. (2018) [84]	X							Across all sites no significant improvements in daily energy purchased.
Olstad et al. (2015) [71]						X		Healthy items represented 7.7%, 22.7%, and 9.8% of sales during the preintervention, intervention, and postintervention periods, with the intervention sales being significantly higher than pre and post. Within the target concession, sales of healthy beverages were equal to or exceeded sales of all other healthy and unhealthy product types.
Pechey et al. (2019) [85]	X							Overall, there were significant reductions in total daily energy purchased from targeted categories.
Reynolds et al. (2021) [89]	X							Total energy purchased significantly declined during the availability intervention (−4.8%), but less than the combined interventions (−11.5%), compared to baseline. Among non-intervention food categories, both intervention periods had less energy purchased (−10%).
Simpson et al. (2018) [90]	X	X						Promotion of total sales from healthier food options increased (+5 percentage points) by late post-intervention. Sales of unhealthy options did not change, except for packaged sweets and chocolates (−5 percentage points by late post-intervention). Changes were not significant.
Tinney et al. (2022) [80]	X	X						Significant increases in the median monthly number of juices and diet drinks sold, while the median weekly number of soft drinks and flavored water decreased. Furthermore, 18% of respondents reported they changed their drink purchases as a result of the SSB removal, 24% adapted by buying SSB elsewhere, and 19% stopped purchasing drinks at the health service.
Velema et al. (2018) [86]	X							Worksite Cafeteria 2.0 resulted in significantly greater purchases for healthier sandwiches, healthier cheese as sandwich fillings, and fruit. No changes occurred for snacks, prepackaged snacks, or “better choice” salads and meat products for sandwiches.
Vermote et al. (2018) [91]	X				X			46% of consumers purchased French fries during baseline week, and 45% during intervention week (no significant difference following portion size reduction).
Wolfenden et al. (2015) [88]						X		Based on intent-to-treat analyses, intervention canteens had significant increases in the proportion of club members who reported purchasing healthy products and non-sugar-sweetened drink purchases.
**(c) Key findings from included studies conducted outside of the U.S. on diet quality outcomes of HFSG interventions (N = 10)**								
**Author (Year)**	**HFSG Setting(s) ^a^**							**Key Findings: Diet Quality**
	**WS**	**HC**	**M**	**CP**	**HE**	**Rec**	**Ch/Y**	
Beer-Borst et al. (2019) [92]	X							Overall, there was a 7% reduction (−0.6 g) in daily salt intake as the primary outcome of interest, but this was not significant after adjusting for sex. Daily salt intake of men declined by 1.2 g (−11.5%) but did not change for women.
Bingham et al. (2012) [94]			X					At 8-week follow-up, the intervention group had greater porridge consumption, lower fruit and berry consumption, and lower fat and fruit and vegetable indices (based on FFQ for conscript populations). At 6 mo, there was greater consumption frequency of fresh vegetables and salad and a higher cereal index.
Geaney et al. (2016) [83]	X							Between baseline and 7–9 months of follow-up for combined vs. control groups, researchers observed significant positive changes in dietary intakes of salt (−1.3 g/day), saturated fat (−7.0 g/day), and energy proportion from saturated fat. Effects in education and environment modifications as single interventions in workplaces were smaller than the combined intervention.
Katz-Shufan et al. (2020) [96]				X				Residents in intervention dining rooms had a significant +0.71 increase in fruit and vegetable portions consumed, while dietary consumption in the control group was unchanged. There were significant increases in mean lunch quality and lunch diversity scores for the intervention group, but not in the control group.
Lassen et al. (2014) [81]	X							Compared to baseline, workers at the intervention site consumed an absolute 17% less energy from fat, a relative 54% more fruits and vegetables, a relative 32% less salt, and a relative 16% less energy in calories (all significant) at follow-up. There were significant increases in the proportion that consumed whole grains and declines in the proportion that consumed refined sugar. Energy consumed at follow-up was significantly higher than at endpoint, although total energy and energy density of meals declined compared to baseline. At the control site, fat consumption significantly increased. There were no changes in whole grain or refined sugar intake.
Seo et al. (2016) [87]				X				Elderly who received nutrition education sessions ate less soup and kimchi after the menus were modified to reduce sodium. All expressed satisfaction with the program, including the different tastes and seasonings.
Uglem et al. (2014) [95]			X					All three baseline intake groups in the intervention camp had significantly higher intake of vegetables, fruits, and semi-whole-grain bread at follow-up than the control camp. In the intervention camp, those with low and medium intake at baseline significantly increased their intake of vegetables, fruits, and semi-whole-grain bread. In the control camp, those with a low intake at baseline significantly increased their intake of vegetables, fruits, and semi-whole-grain bread. Those with a medium intake at baseline did not have significant changes at follow-up, and those with a high intake at baseline had a significant reduction in intake at follow-up.
Vanderlee et al. (2014) [74]	X	X						Compared to the control site, patrons at the intervention site consumed significantly less energy (−21%), sodium (−23%), saturated fat (−33%), and total fat (−37%).
Vermote et al. (2018) [91]	X				X			Significant differences in total consumption of French fries following portion size reduction (−9%).
Vitale et al. (2018) [93]	X							Significantly more employees met recommendations for saturated fat, cholesterol, sugar, and fiber intake after intervention. At 6-month follow-up, there was significantly higher consumption of whole-grain dishes, legumes, and white meat/fish and reduced consumption of refined carbs, processed meat, eggs, and cheese. At 3-yyear follow-up, there was some attenuation, but healthy changes to consumption for whole-grain pasta, legumes, vegetables/fruit, white meat, refined carbs, red/processed meat, eggs, and cheese remained significant.
**(d) Key findings from included studies conducted outside of the U.S. on health outcomes of HFSG interventions (N = 2)**								
**Author (Year)**	**HFSG Setting(s) ^a^**							**Key Findings: Health**
	**WS**	**HC**	**M**	**CP**	**HE**	**Rec**	**Ch/Y**	
Geaney et al. (2016) [83]; Fitzgerald et al. (2018) [33]	X							Between baseline and 7–9 mo post-implementation, the intervention group that received both environmental dietary changes and nutrition education had a significant decrease in body mass index (−1.2 kg/m^2^).
	X							All intervention components delivered improvements to QALY, but improvements were greatest in the environmental group (+0.05 QALY), followed by the education and combined groups. The control group had deterioration in health (−0.01 QALY).
**(e) Key findings from included studies conducted outside of the U.S. on financial implications of HFSG interventions (N = 10)**								
**Author (Year)**	**HFSG Setting(s) ^a^**							**Key Findings: Financial Implications**
	**WS**	**HC**	**M**	**CP**	**HE**	**Rec**	**Ch/Y**	
Boelsen-Robinson et al. (2017) [76]	X	X						Because the health service was able to increase commission percentages on vended items pre-implementation, the decline in sales of red, less-green, and green drinks did not affect revenue.
Boelsen-Robinson et al. (2020) [82]						X		At the end of the intervention and 1-year follow-up, there was a 25% decline in all cold drink sales compared to predicted sales without the intervention.
Di Sebastiano et al. (2020) [68]					X			Across all three dining halls (both intervention and control), there was a 12–24% decline in total revenue corresponding to a 20–26% decline in units sold. The greatest decline in revenue was in the semester immediately prior to the Healthy Beverage Initiative implementation.
Fitzgerald et al. (2018) [33]	X							Cost utility: Costs per employee were the least in the control condition, followed by environmental dietary modification, nutrition education intervention, and combined intervention. Cost benefit revealed the most improvement in absenteeism in the combined intervention (−0.78 days), then environmental dietary modification, then nutrition education intervention; there was increased absenteeism in the control (+0.34 days).
Katz-Shufan et al. (2022) [34]				X				The mean incremental cost per serving of nutritionally improved recipes was USD 0.11.
Olstad et al. (2015) [71]						X		The proportion of total revenues per patron did not change.
Pechey et al. (2019) [85]	X							No evidence of revenue impact.
Reynolds et al. (2021) [89]	X							Revenue declined during availability intervention (−2.6%) and more so during combined interventions (−5.7%).
Simpson et al. (2018) [90]	X	X						Total sales increased at early post-intervention (+11%) and through late post-intervention (+27%).
Wolfenden et al. (2015) [88]						X		Based on intent-to-treat analyses, intervention had no effect on club annual revenue.

HFSG: healthy food service guidelines; SSB: sugar-sweetened beverage; FFQ: food frequency questionnaire; QALY: quality-adjusted life years. Underline indicates primary HFSG intervention setting. ^a^ HFSG settings are abbreviated using WS (worksite), HC (health care), M (military), CP (community programs), HE (higher education), Rec (recreation centers), and Ch/Y (child and youth programs). Some studies conducted interventions that might overlap with two settings, such as a hospital worksite cafeteria. Because the study population was hospital and health care workers, these studies would be categorized as having occurred in the worksite. Community program settings include congregate meal programs, public zoo facilities, parks, and other food service venues in institutions or programs that serve the public. ^b^ Lane et al. (2019) [35] was a sub-study that used data from the HFSG evaluation conducted in Olstad et al. (2019) [72], so the two publications are presented together as representing one unique intervention.

## 4. Discussion

This scoping review characterized the landscape of peer-reviewed primary literature that assessed food environment, diet quality, health, and financial outcomes of HFSG interventions in worksites, (non-school) institutions, and community settings. This literature has grown considerably since the most recent comparable systematic review published in 2014 [15], which identified nine peer-reviewed evaluations of healthy food procurement policies in non-school settings. Among 68 included papers, this review summarized the range of food service settings and venues, study designs, intervention details, outcome measures, findings, and other characteristics in U.S. and non-U.S. contexts. Our broad inclusion criteria resulted in a rich dataset of HFSG interventions that have been implemented and evaluated across the world in heterogeneous settings and populations. Having documented a wide array of interventions and outcomes, this review can inform institutional leaders and food service operators seeking to implement HFSG.

### 4.1. Critical Gaps in the Literature

An objective of this review was to identify gaps in the literature that indicate where additional evidence could (1) help demonstrate the impact of HFSG to institutional decision-makers and (2) improve intervention effectiveness. The body of evidence—both in and outside the U.S.—that met our inclusion criteria was larger than anticipated, with most of the interventions targeting food service venues where food is sold, most commonly in worksite settings. We found that venues where food is served are generally understudied in comparison. In settings where food is served, like prisons or shelters, the population is typically partially or totally dependent on the institution for meeting nutritional needs. Additionally, these populations may have higher risk and prevalence of diet-related chronic diseases [97,98,99]. The lack of research in correctional facilities may be due to unique ethical requirements for research involving incarcerated people [100]. Studying the effects of HFSG in settings where food is served could guide future decisions regarding HFSG implementation in populations under the care of publicly funded institutions.

Among settings where food is sold, we identified only five studies that included higher education settings [39,53,65,68,91]. Since it is common for students at U.S. universities to live in campus housing where a meal plan provides a high proportion of their daily meals, U.S. higher education settings are an important area for HFSG intervention and evaluation.

Regarding the outcomes of interest, food environment outcomes were reported most often (57%), followed by consumer behavior and financial implications (34% for both), diet quality (24%), and health outcomes (9%). The prevalence of each outcome category appears to be associated with ease of measurement, with the more downstream outcomes (diet quality and health) more challenging and resource-intensive to measure. Most of the studies including measures of health outcomes were modeling simulations, which use estimated inputs and logic-based assumptions to generate potential health impacts. Unfortunately, no RCTs were conducted to measure health outcomes. Such RCTs would provide external validity to simulation studies’ results that generally support HSFG as promoters of health, as simulations rely on assumptions of HSFG implementation fidelity and effectiveness. The ultimate goals of HFSG are generally to improve diet quality and health. Therefore, quasi-experimental evaluations of the effects of HFSG on diet quality and short- to medium-term risk factors for cardiometabolic disease (weight, blood pressure, hemoglobin A1c, etc.) or long-term disease outcomes (diabetes and hypertension, cardiovascular events, deaths from diet-related diseases, etc.) would strengthen the literature available to institutional decision-makers and to practitioners.

Evidence synthesis was beyond the purview of this scoping review, but it would be necessary to draw conclusions about the effectiveness of various types of HFSG interventions to better inform policy and practice. Based on our findings, there likely are a sufficient number of studies that assessed food environment, consumer behavior, and financial implications to conduct separate systematic reviews on the effects of HFSG interventions. In contrast, there is likely insufficient evidence at this stage to synthesize and draw firm conclusions on the effects of HFSG on diet quality and health outcomes. Additionally, our findings point to subsets of HFSG interventions that may have been studied extensively enough to enable evidence synthesis, including those targeting sodium and SSB reduction. Finally, using study designs that allow outcomes to be attributable to HFSG interventions (e.g., pre-post with control, time series, and RCT) will facilitate comparability and synthesis across evaluations, as well as assessment of evidence quality.

### 4.2. Strengths and Limitations

To our knowledge, this review is the first to scope research on HFSG in only non-school, non-ECE institutions, worksites, and community settings. We followed PRISMA-ScR guidelines, and two authors independently reviewed all papers for inclusion or exclusion at the full-text stage. Due to resource limitations, only one author independently screened all papers at the title and abstract stage. We mitigated this limitation by having two authors conduct a pilot screening to reach strong agreement for inclusion and exclusion on a subset of search results. Similarly, although only one author independently extracted data from all included studies, a second author verified the accuracy and completeness of all extracted data.

Moreover, due to resource limitations, we were unable to search for studies published in languages other than English, which may have biased our dataset in selecting studies conducted in high-income countries, particularly the U.S., Canada, the U.K., Europe, and Australia. Stakeholders who are interested in developing evidence-based HFSG interventions in low- and middle-income countries may need to scope publications in other languages to more definitively identify research gaps in those contexts.

Another limitation is that 40% of our included studies were identified through snowball searching, indicating that our primary search strategy did not capture all papers of interest. There may be additional publications that we did not find that would have met our inclusion criteria. One reason for this may be that the studies of interest used a wide range of terminology to describe their interventions; few used HFSG per se. Pilot searches using MeSH terms excluded many studies that we had identified a priori as meeting inclusion criteria, so we prioritized searching keywords, titles, and abstracts to identify the broadest filter for the database searches. Even so, studies retrieved through the search strategy had limited terminology overlap with studies identified through snowball searching; only 33 (18%) of 184 unique keywords used across all HFSG interventions were shared among studies retrieved through the two approaches. More consistent terminology in this area of research—like using the standardized HFSG outcome definitions and intervention settings presented in this review as keywords for peer-reviewed publications—could facilitate comprehensive evidence scoping and synthesis.

There may be evaluations of HFSG interventions that have been published outside the peer-review process and were unavailable to be retrieved for this analysis. Future reviews could scope the gray literature to capture evaluations not published in peer-reviewed journals that could add to our knowledge of HFSG interventions and their outcomes. Examples include pragmatic evaluations of HFSG policies conducted in places like New York City and Washington State (U.S.) [101,102]. Furthermore, since our systematic search was completed in 2022, a small number of studies have been published that would meet our inclusion criteria. The studies include a long-term follow-up to the implementation of a healthy food policy in New Zealand hospitals [103] and an HSFG intervention in hospital cafeterias in Vancouver, Canada [104]. This growing body of literature supports our discussion of new opportunities for systematically assessing the quality of evidence for HSFG as a lever for improving public health nutrition.

## 5. Conclusions

This scoping review characterized a growing body of evidence on the effectiveness of HFSG interventions to improve the food environment, consumer behavior, diet quality, and health in worksites, institutions, and community settings, as well as short- and long-term financial implications. This review offers a starting point for researchers, practitioners, and policymakers to understand the range of existing HFSG interventions and their outcomes. The majority of HFSG interventions took place in venues where food is sold (e.g., worksite cafeterias, vending machines). A diversity of HFSG terminology and measurement tools demonstrates the literature’s breadth. Literature gaps include quasi-experimental designs, as well as interventions in settings that serve dependent populations (e.g., universities, elderly feeding programs, and prisons).

## Figures and Tables

**Table 1:** Descriptive characteristics of included studies by study location, N (%).

Characteristic	All Studies N = 68 (100.0)	United States N = 34 (50.0)	Outside the United States N = 34 (50.0)
**Study Design**			
Cross-sectional	5 (7.4)	3 (8.8)	2 (5.9)
Pre-post (without control)	31 (45.6)	18 (52.9)	13 (38.2)
Pre-post (with control)	16 (23.5)	5 (14.7)	11 (32.4)
RCT	8 (11.8)	2 (5.9)	6 (17.6)
Other ^a^	8 (11.8)	6 (17.6)	2 (5.9)
**HFSG Intervention Setting**			
Workplace	36 (52.9)	17 (50.0)	19 (55.9)
Health care	23 (33.8)	13 (38.2)	10 (29.4)
Military	6 (8.8)	4 (11.8)	2 (5.9)
Community program	16 (23.5)	13 (38.2)	3 (8.8)
Higher education	5 (7.4)	3 (8.8)	2 (5.9)
Recreation center	11 (16.2)	3 (8.8)	8 (23.5)
Child/youth out-of-school program	4 (5.9)	4 (11.8)	0 (0.0)
**HFSG Intervention Venue**			
Where foods are sold ^b^	60 (88.2)	27 (79.4)	33 (97.1)
Vending machines	28 (41.2)	15 (44.1)	13 (38.2)
Cafeterias, canteens, and dining halls	41 (60.3)	19 (55.9)	22 (64.7)
Retail outlets ^c^	27 (39.7)	14 (41.2)	13 (38.2)
Where foods are served ^d^	12 (17.6)	11 (32.4)	1 (2.9)
Non-therapeutic patient meals	3 (4.4)	3 (8.8)	0 (0.0)
**HFSG Outcome Type ^e^**			
Food environment	39 (57.4)	21 (61.8)	18 (52.9)
Consumer behavior	23 (33.8)	10 (29.4)	13 (38.2)
Diet quality	16 (23.5)	6 (17.6)	10 (29.4)
Health	6 (8.8)	4 (11.8)	2 (5.9)
Financial implications	23 (33.8)	13 (38.2)	10 (29.4)

Notes: We use the following abbreviations: HFSG (healthy food service guidelines), RCT (randomized controlled trial). ^a^ “Other” study designs include the following: simulation studies; studies that assessed outcomes at two points in time, one of which could not be attributable to or associated with the HFSG intervention (e.g., Donohoe, Mather, and McGurk, 2014 [36]); reversal intervention design (e.g., Karpyn et al., 2020 [37]); and studies that used longitudinal/repeated measures (e.g., Losby, 2014 [38]; Yarnoff, 2022 [39]). ^b^ Food service venues in which food is sold include cafeterias, cafes, and vending machines. Customers purchase their food and beverages from a variety of choices. ^c^ Retail outlets are distinct from cafeterias, canteens, and dining halls where customers also purchase their food and beverages from a variety of choices; they refer only to shops, kiosks, concessions, and other small commercial venues that are not standalone (e.g., brick-and-mortar restaurants). ^d^ Food service venues in which food is served include hospitals (non-therapeutic patient meals), congregate meal programs, shelters, and correctional facilities. The institution is responsible for providing people with meals that meet all or part of the daily or weekly nutrition needs, and individual choice may be limited. ^e^ The outcomes were defined by the authors as follows. Food environment: availability of healthier or less healthy offerings regarding what is served or offered for sale (e.g., modifying ingredients or recipes, adding or removing menu items, and making healthier choices the default). Consumer behavior: selections or sales of healthier or less healthy offerings in venues where the consumer has a choice of multiple options. Diet quality: consumption of healthier or less healthy items, intake of key food groups, nutrients, or food components (e.g., vegetables, fiber, and sodium), or alignment with a holistic diet quality index. Health: anthropometric or biochemical indicators of diet-related chronic disease risk (e.g., weight and blood pressure) and diet-related chronic disease morbidity or mortality (e.g., projected incidence of diabetes). Financial implications: short- and long-term financial consequences associated with the HFSG intervention (e.g., implementation costs, changes in overall revenue of the food service operation, and projected health care cost savings). Percentages may not sum to 100% due to some studies measuring multiple outcomes.

**Table 2:** Descriptive information for included studies in the United States (N = 34) representing 32 unique interventions.

Citation	Author (Year)	Study Objective	Location	Study Design	Intervention Description
[42]	Abrahams-Gessel et al. (2022)	Estimate the health impact and cost-effectiveness of federal food service guideline implementation at worksite cafeterias.	National	Microsimulation (5 yrs and lifetime horizons)	Simulated the effects of implementing federal food service guidelines in government and large corporate cafeterias.
[51]	Basu et al. (2020)	Study the cost-effectiveness of a workplace sugar-sweetened beverage (SSB) sales ban in California-based health care organizations on employee health and health care spending.	California	Microsimulation (10 yrs and lifetime horizons)	The removal of SSB from cafeterias, vending machines, and other retail outlets was estimated to reduce SSB consumption by 1.5 oz/person/day. This measure was used to simulate the impact of reduced SSB consumption.
[55]	Belanger and Kwon (2016)	Evaluate the impact of partial implementation of initial military training (IMT) menu standards in a non-trainee dining facility on food selection and nutrient intake of soldiers.	Midwest	Pre-post, no control (3 wks)	Lunch and dinner menus altered according to IMT standards (increase lower-calorie, nutrient-dense selections; limit choices with poor nutrition). Nutrition quality was indicated to diners using the Army’s “Go for Green” labeling system (green labels are optimal choices).
[56]	Berkowitz et al. (2016)	Determine the impact of providing reduced-size entrée options on food selection, energy, and nutrient intake at the worksite cafeteria.	Minneapolis/St. Paul, Minnesota	Pre-post, no control (7 wks)	Six entrees (meatloaf, spaghetti, pork loin, lasagna, chopped steak, and chicken parmesan) were available as full- and reduced-size options.
[57]	Brooks et al. (2017)	Reduce the percentage of higher-sodium prepackaged food products in community institutions serving or located in Black and Latine communities with disproportionate rates of chronic disease.	Boston, Massachusetts	Pre-post, no control (1–1.5 yrs by site)	Reduce availability of prepackaged foods (food facings) at access points (vending machines, cafeterias/kiosks) with ≥200 mg sodium/serving.
[58]	Cole et al. (2018)	Assess the effectiveness of performance-based nutrition standards informed by the 2010 Dietary Guidelines for Americans (DGA) on patron diet quality.	Fort Bragg, North Carolina	Pre-post, with control (12 mo)	Performance-based nutrition standards increased availability/use of new high-quality, nutrient-dense foods (Greek yogurt, walnuts, kale, whole grains). Reduced saturated fats in food preparation for all meals.
[48]	Cradock et al. (2015)	Evaluate the change in access to healthy beverages in Boston city agencies following implementation of the Healthy Beverage Executive Order.	Boston, Massachusetts	Pre-post, no control (2 yrs)	Eliminated SSB sales and marketing in vending machines and city-managed food/beverage service programs and established nutrition standards for beverages offered for sale.
[59]	Cradock et al. (2022)	Evaluate the impact of evidence-based nutrition guidelines for sugar and sodium for prepackaged and prepared food and beverage facings.	Massachusetts	Pre-post, no control (2 yrs)	Reduced “red” (drink rarely, if at all) beverage availability. Reduced sodium in packaged and prepared foods according to established nutrition guidelines and state policies. “Low sodium” is considered for packaged snacks (≤200 mg); cafeteria plates, entrees, deli, and grill items (≤805 mg); and side dishes/soups (≤480 mg). Strategies were tailored to each health care setting’s capacity to support implementation within the cafeteria and vending.
[60]	Crombie et al. (2013)	Test the feasibility and efficacy of an intervention to improve nutritional intake in a military dining facility.	Fort Bragg, North Carolina	Crossover RCT (6 mo + 6 mo)	Changed dining facility serving practices to align jointly with the 2005 DGA and Army Regulation 40–25, e.g., increased availability of fresh fruit/produce on serving lines and reduced availability of foods high in dietary fat and sugar. Increased healthy cooking methods for main entrees. Staff training on cooking methods and portion standardization.
[36]	Donohoe Mather and McGurk (2014)	Outline preliminary findings and impact evaluation plans for the Choose Healthy Now! healthy vending pilot project.	O’ahu, Hawai’i	Other (baseline [assessed prior to official launch] vs. pre [at public launch])	Increased inventory of healthy items in worksite cafeterias and snack shops based on a traffic light nutrition coding system using simplified labels (green: go, yellow: slow, red: uh-oh).
[61]	Durant et al. (2020)	Evaluate the impact of healthy procurement and serving practices in community settings on community residents’ behaviors regarding sugary beverages.	Upstate New York counties	Pre-post, with control (1 yr)	Limited availability of sugary beverages in childcare centers, school districts, after-school programs, hospitals, and some private companies via promotion of vending practice and wellness policy changes for healthier vending standards.
[52]	Eneli et al. (2014)	Report changes in beverage consumption and sales revenue following the organizational SSB ban, part of the strategic goals of the National Children’s Hospital Wellness Initiative.	Columbus, Ohio	Pre-post, no control (1 mo)	Removed SSB from all hospital-owned (hospital cafeteria, a food court, coffee shop, and two gift shops) and contracted venues (including vending machines). Only milk products and diet varieties of carbonated beverages, energy drinks, and 100% fruit juices were allowed.
[53]	Epel et al. (2020)	Test the association between the SSB sales ban and SSB intake, abdominal adiposity, and insulin sensitivity among heavy SSB drinkers within the employee population.	San Francisco, California	Pre-post (10 mo)	Eliminated the sale of SSB in all venues, including cafeterias, vending machines, hospital food services, and retail outlets. Half of the study participants were randomized to receive a brief 2-month motivational intervention targeting reductions in SSB intake.
[40]	Hanson et al. (2020)	Evaluate the U.S. Army Child, Youth, and School Healthy Menu Initiative’s impact on diet quality.	Continental U.S. and U.S. facilities in Europe	Pre-post, no control (approx. 3 yrs)	Implemented standardized, seasonal, cost-effective menus compliant with the 2015–2020 DGA and 2017 Child and Adult Care Food Program rules and best practice recommendations.
[62]	Hansotte et al. (2021)	Evaluate the effectiveness of recipe modification through speed-scratch cooking on overall sodium levels in meals provided through congregate or home-delivery programs.	Marion County, Indiana	Pre-post, with mock control (1 yr)	Reduced sodium content of two potato dishes to meet Family and Social Services Administration standards. Mashed potatoes: used unseasoned potato pearls and low-sodium flavor base. Scalloped potatoes: used reduced-sodium sauce in cooking.
[44]	Hopkins et al. (2012)	Process the evaluation of the intervention Working Out Regularly Keeps Individuals Nurtured and Going.	Los Angeles County, California	RCT (6 mo)	Provided training and resources for establishing healthy food procurement for catering/conference facility menus; providing ≥50% healthy, competitively priced food options in vending, cafeterias, and on-site retail; and including language that mandates/incentivizes healthy procurement in subcontracts.
[43]	Jilcott Pitts et al. (2016)	Examine barriers, facilitators, costs, and profitability related to implementing food service guidelines in federal worksite and hospital cafeterias.	National	Cross-sectional	Federal worksites adhered to the Health and Sustainability Guidelines for Federal Concessions and Vending Operations; hospitals adhered to the Partnership for a Healthier America Hospital Healthier Food Initiative; both guidelines prioritized procurement, preparation, and offering of healthy foods and beverages that limit trans fat, sodium, and added sugars.
[41]	Jordan et al. (2020)	Evaluate the success of CDC’s Sodium Reduction in Communities Program (SRCP) regarding increasing access, availability, and purchase of reduced-sodium foods.	National	Pre-post, no control (2–3 yrs)	Strategies included developing and implementing food service guidelines and nutrition standards, implementing menu and recipe modifications to reduce sodium, and implementing strategies to enhance the selection or purchase of low-sodium foods.
[37]	Karpyn et al. (2020)	Assess the impact, on sales, of introducing healthier items (with animal character marketing) at a zoo concession stand.	Unnamed urban community in Mid-Atlantic U.S.	Reversal (ABABABAB, 8 wks)	Introduced seven new healthy food items: fruits, vegetables, non-fat/low-fat dairy, and packaged items (considered healthy if they meet Tier 1 or 2 of the Nutrition Environment Measures Survey-Vending criteria).
[63]	Laroche et al. (2014)	Evaluate the effect of healthy concession intervention on sales of products and concession revenue at a high school.	Muscatine, Iowa	Pre-post, no control (1 yr)	Modified menus to introduce eight healthier foods that aligned with 2007 U.S. Department of Agriculture Team Nutrition guidelines for competitive foods (<35% total fat, <10% saturated fat, ≤35% sugar by weight). Eliminated trans fats in cooking oil and introduced fruits and vegetables.
[64]	Lessard et al. (2014)	Assess the impact of the healthful food and beverage vending program on the availability and purchases of new healthy items and site revenue.	Delaware	Pre-post, no control (6 mo)	≥75% of items must meet the local pediatric health system’s guidelines for Go/Slow. “Go”/”Slow” foods: ≤200 cal, 35% cal from fat, 10% cal from sat fat, 200 mg sodium, no trans fats or candy. “Go” beverages: unsweetened water. “Slow” beverages: 100% juice or ≤10 cal/8 oz serving. All other foods are “Whoa.”
[65]	Lillehoj et al. (2015)	Identify implementation successes of increasing availability of healthier food and beverage vending choices.	Iowa	Pre-post, no control (1 yr)	Aimed to increase green- and yellow-coded foods, based on Institute of Medicine standards, modified based on the Iowa Healthy Kids Act: Foods and beverages must have ≥1 serving of fruit, vegetable, whole grains, or non-low-fat dairy products and meet the 2005 DGA.
[29,52]	Long et al. (2018); Long et al. (2021) ^a^	Describe outcomes of the SRCP project in venues serving populations (Pacific Islander, low-income, food-insecure) at elevated risk for hypertension.	Northwest Arkansas	Pre-post, no control (10–11 mo)	Comprehensive food service guidelines that included sodium reduction aimed to create standardized food purchasing lists, implemented food preparation practices to reduce sodium, and developed recipes for lower-sodium menu items that could still incorporate higher-sodium restaurant-donated foods.
				Pre-post, no control (~3 yr)	Created a standardized food purchasing list, including lower-sodium alternatives (not achieved in Long et al., 2018 [54]). Continued with modified, lower-sodium menu items.
[38]	Losby et al. (2014)	Summarize the preliminary impact of sodium-reduction strategies in programs serving meals to older adults.	Broome, Schenectady Counties, New York	Longitudinal, no control (Broome: 1 yr Schenectady: 2 yrs)	Broome: aimed for a 10% reduction in average sodium content in meals over 2 years. Schenectady: aimed for 30% reduction over 3 years. Primary strategies were product substitutions, recipe modifications, and scratch cooking.
[30,46]	Moran et al. (2015); Moran et al. (2016) ^b^	Describe the nutritional quality of regular-diet patient menus before and after the Healthy Hospital Food Initiative (HHFI).	New York City, New York	Pre-post, no control (3.1–28.4 mo, median 8 mo)	HHFI nutrition standards aligned with 2010 DGA and Dietary Reference Intakes aimed to improve the nutrient profile of menus (e.g., limit sodium and increase fiber) and select foods for evidence-based disease prevention (e.g., fruits, vegetables, and whole grains).
		Describe the process and assess key outcomes of HHFI implementation in patient meals, beverage and food vending, and cafeterias/cafes.		Pre-post, no control (baseline: Jan 2012–June 2014; endline: July 2014–Sept 2014)	Patient meals: sodium limits for cereals, guidelines for meals served (five daily fruit and vegetable servings). Beverage vending: ≤2 sugary drink slots, limited portion sizes and ads for sugary drinks, andwater at eye level. Food vending: nutrient requirements per package, promoted whole foods over grain-based snacks. Cafeterias/cafes: 20 total criteria to limit sodium, sugary drinks, and calorie-dense foods while promoting water, fruits, vegetables, and whole grains.
[50]	Narain et al. (2016)	Examine the effect of a healthy vending policy on beverage quality in municipal parks.	Carson, California	Pre-post, post-only control (6 mo)	The Carson resolution required all beverages sold/distributed at city parks to be low in fat, calories, sugar, and sodium. Used the Nutrition Environment Measures Survey-Vending traffic light system to categorize vending items.
[49]	Pharis et al. (2017)	Assess the impact of healthy snack and beverage vending standards on sales of healthy and less healthy items and total sales volume.	Philadelphia, Pennsylvania	Pre-post, no control (1–2 yrs)	Vending contracts required two-thirds of items to meet healthy nutrition standards based on the 2010 DGA and downsize from 20 oz to ≤12 oz sugary drink portions.
[66]	Ranke et al. (2015)	Assess whether hospitals that are part of the Balanced Menus Challenge (BMC)—using both a climate change mitigation strategy and an effort to bring healthier meat into health care settings to preserve antibiotic effectiveness and promote good nutrition—reduced meat purchasing.	Maryland and Washington, DC	Cross-sectional	BMC guided hospitals to reduce meat purchasing by 20% and invest cost savings into buying locally produced meat or meat with a smaller environmental footprint. Strategies: eliminate/reduce meat-containing meals, use meat as a condiment to meals, reduce portions, purchase fewer/cheaper cuts, and increase vegetarian proteins.
[47]	Volger et al. (2022)	Examine the association between voluntary portion size restriction on SSB and the volume of SSB purchased and consumed and food calories purchased during basketball games at a sports arena (Barclays Center).	New York City, New York	Cross-sectional, with control	Barclays Center voluntarily adopted a maximum 16 oz SSB portion size cup.
[45]	Wickramasekaran et al. (2018)	Evaluate adherence to a 100% healthy vending policy and measure change in nutritional content of products and revenue.	Los Angeles County, California	Pre-post, no control (2 yrs)	Individually sold snacks could not exceed 250 cal, 360 mg sodium, 35% sugar by weight, 10% cal from saturated fat, and 35% cal from fat. Beverages could include only drinking/carbonated water, ≥50% fruit juice drinks without added sweetener, unsweetened milk products, sugar-sweetened or artificially sweetened drinks ≤25 cal/8 oz, and bottled water priced lower than the highest other beverage.
[67]	Yan et al. (2019)	Assess the impact of a worksite 100% healthy vending model on revenue and nutritional quality of products sold.	Dallas, Texas	Pre-post, no control (1–2 yrs)	Serving standards according to the American Heart Association Healthy Workplace Food and Beverage Toolkit. Food: ≤200 cal, ≤240 mg sodium, 0 g trans fats, ≤1 g saturated fat, no candy or fried chips. Nuts/fruit mixes: ≤1.5 oz, ≤140 mg sodium. Beverages: water ≤10 cal, low/fat-free milk or alternatives ≤130 cal/8 oz, 100% fruit/vegetable juice without added sugar and ≤120 cal/8 oz, and other beverages ≤10 cal.
[39]	Yarnoff et al. (2022)	Estimate the cost of achieving SRCP implementation outcomes and the cost-effectiveness of SRCP implementation strategies.	National	Longitudinal (cost), simulation (cost-effectiveness)	Strategies included sodium-related food service guidelines and nutritional standards; meal/menu modifications; and lower-sodium food procurement practices.

HFSG: healthy food service guidelines; RCT: randomized controlled trial. ^a^ These separate publications evaluated the same HFSG program (Sodium Reduction in Communities Program [SRCP]), so they are presented together as one unique intervention. Long et al. (2021) [54] was a longer-term follow-up to the study initially evaluated in Long et al., 2018 [29]. ^b^ These separate publications evaluated the same HFSG program (Healthy Hospital Food Initiative [HHFI]), so they are presented together as one unique intervention. Moran et al. (2015) [46] assessed non-therapeutic patient menus, while Moran et al. (2016) [30] was a broader evaluation of HHFI in other hospital venues.

**Table 3:** Key findings from included studies (United States).

(a) Key Findings from Included Studies (United States) on Food Environment Outcomes of HFSG Interventions (N = 21)								
Author (Year)	HFSG Setting(s) ^a^							Key Findings: Food Environment
	WS	HC	M	CP	HE	Rec	Ch/Y	
Belanger and Kwon (2016) [55]			X					Meal offerings were significantly different from baseline to intervention. Using the traffic light paradigm to indicate healthfulness, at baseline 39% were red, and 41% were green (optimal). During the intervention, 17% were red and 61% were green.
Brooks et al. (2017) [57]		X		X		X		Overall, there was a 7.5 percentage point decline in the proportion of prepackaged snack facings with ≥200 mg sodium/serving. When stratified by access point type, the only significant declines were in cafeterias/kiosks (not vending machines). Vending machine access points had significant declines in YMCAs, but not community health centers, organizations serving homeless populations, or hospitals.
Cradock et al. (2015) [48]	X			X				Average calories and sugar content per beverage decreased following Healthy Beverage Executive Order implementation. Prices did not change over time. Availability of high-sugar beverages declined while low-sugar beverage availability increased. City agencies had 4.9 times the odds of selling only low-sugar beverages compared to pre-implementation.
Cradock et al. (2022) [59]	X	X						At follow-up, unsweetened and low-sugar beverages were significantly more prevalent in vending machines and cafeterias. Low-sodium packaged foods and prepared foods were more prevalent in cafeterias, but not vending machines.
Donohoe Mather and McGlurk (2014) [36]	X							At baseline, 7% of items from vendors were green (optimal), and 21% were yellow. By the official launch of the pilot project, vendors reported an increase in the number of green products by 128% and yellow products by 10%.
Durant et al. (2020) [61]		X		X			X	Based on difference-in-difference analyses, availability of regular soda declined in intervention counties.
Hanson et al. (2020) [40]			X				X	The post-initiative menu achieved higher Healthy Eating Index (HEI) 2015 scores overall and for components (total vegetables, fatty acids, and greens/beans). Pre- and post-initiative menus both scored highly on the dairy and whole fruit components and low on whole grains.
Hansotte et al. (2021) [62]				X				Reduction in sodium content of scalloped potatoes (65%) and mashed potatoes (87%). Reduction of 12% for average sodium per meal in both home-delivered and congregate meals.
Hopkins et al. (2012) [44]	X	X						In this study, three of fifteen sites were identified as “model adaptors” that implemented and sustained core elements (increased availability of healthy snacks) with minor lapses in activities. It is critical to involve top-level organizational leaders and middle management decision-makers for successful long-term implementation.
Jilcott Pitts et al. (2016) [43]	X	X						All sites added healthier items, purchased more fresh produce, and modified recipes according to guidelines. Aligning values with clients, consumers, and vendors facilitated guideline implementation. Challenges arose from needing more staff and time to properly implement new food service measures and receiving negative consumer feedback. Challenges at federal worksites included purchasing 100% juice and higher-fiber + lower-sugar cereals and removing fryers and deep-fried products. Challenges at hospitals included removing salt and frying for food preparation.
Jordan et al. (2020) [41]	X	X		X				Worksites: +81 orgs offering new low-sodium foods, −44 mg avg sodium content of targeted foods. Hospitals: +39 orgs offering new foods, −223 mg avg sodium content. Congregate meals: +91 orgs offering new foods, −386 mg avg sodium content.
Lessard et al. (2014) [64]	X							All sites eventually reached full compliance with the healthy vending pilot, but healthful beverage standards took longer to meet than healthful food standards. Time to compliance ranged from 6 to 19 weeks across the three sites.
Lillehoj et al. (2015) [65]	X				X			The majority of vending options did not meet the criteria for improved healthfulness. Only the county government worksite had a machine that increased healthy offerings (15% to 26%). The college site had decreased offerings (8% to 6%).
Long et al. (2018) [54]; Long et al. (2021) [29] ^b^				X				In this study, 4% (6) of the recipes were modified to reduce sodium content. Mean sodium content of meals offered declined 38% (1710 to 1053 mg). Mean sodium served per diner decreased 17% (1509 to 1258 mg) from baseline to follow-up.
				X				From baseline to year 3: mean sodium served per diner decreased 36% (1443 to 944 mg), mean energy served per diner decreased 23% (621 to 479 kcal), and mean sodium served per 1000 kcal per diner decreased 16% (2397 to 2025 mg).
Losby et al. (2014) [38]				X				Broome County (after 1 year): 16% reduction (1517 to 1266 mg) in sodium per congregate meal, 16% reduction (1163 to 975 mg) in sodium per home-delivered meal. Schenectady County: 10% reduction (1270 to 1146 mg) per meal in year 1, 14% reduction (1379 to 1184 mg) per meal in year 2.
Moran et al. (2015) [46]; Moran et al. (2016) [30] ^c^		X						At baseline, no hospital’s regular-diet menu met all Healthy Hospital Food Initiative standards. After implementation, key standards were met: a 25% increase in fiber, a 19% decrease in sodium, a 24% decrease in calories from fat, a 21% decrease in calories from saturated fat, a 667% increase in fresh fruit servings, and decreases in full/reduced-fat milk servings (100%), refined grains (35%), and frequency of desserts (92%).
		X						In this study, 12% of public hospitals implemented cafeteria/café standards. Among private hospitals, 71% met standards for patient meals, 58% for beverage vending, 50% for food vending, and 67% for cafeterias/cafes. Most private hospital cafeterias introduced healthy value meals, removed unhealthy items from entrances and points of purchase, increased whole grain availability, and reduced pastry/dessert calorie density.
Narain et al. (2016) [50]				X				Before policy implementation, the mean proportions of green (optimal), yellow, and red items were 16%, 14%, and 70%. After policy implementation, mean proportions were 21%, 70%, and 8%.
Ranke et al. (2015) [66]		X						Three out of six hospitals tracked their progress and reduced red meat purchases. Three hospitals increased/substituted poultry and fish and increased vegetarian options or proteins. The most common strategy used to decrease meat purchasing was one-to-one substitution.
Wickramasekaran et al. (2018) [45]	X			X				At baseline, approximately 35% of the snacks and 51% of the beverages stocked in vending machines adhered to the 100% healthy vending policy. By the end, the adherence of vending offerings was 61% (snacks) and 98% (beverages).
**(b) Key findings from included studies (United States) on consumer behavior outcomes of HFSG interventions (N = 10)**								
**Author (Year)**	**HFSG Setting(s) ^a^**							**Key Findings: Consumer Behavior**
	**WS**	**HC**	**M**	**CP**	**HE**	**Rec**	**Ch/Y**	
Belanger and Kwon (2016) [55]			X					Using a traffic-light-style classification system, the percentage of red-labeled items selected was lower (45% vs. 18%) and green-labeled items higher (36% vs. 58%) after the intervention compared to baseline.
Berkowitz et al. (2016) [56]	X							Selection of reduced-size entrées increased over the 7-week study period.
Jordan et al. (2020) [41]	X	X		X				All sites observed an increased number of people purchasing or selecting low-sodium foods and the number of low-sodium food items sold from baseline to follow-up.
Karpyn et al. (2020) [37]				X				Healthy items sold more frequently during weeks when animal cartoon characters were displayed, although they were consistently less popular than unhealthy items.
Laroche et al. (2014) [63]							X	Gradual increases in purchases of string cheese, apples, and carrots in 2009 from 2008.
Lessard et al. (2014) [64]	X							Purchases of healthiest (“Go”) and healthier (“Slow”) increased over the course of the pilot at some sites.
Pharis et al. (2017) [49]	X			X		X		Post-conversion, 40% of snack sales and 46% of beverage sales are attributable to healthy items. Healthy snack (323%) and beverage (33%) sales increased from baseline and during the conversion period, respectively.
Volger et al. (2022) [47]						X		For all eventgoers, a 16 oz SSB portion size cap was associated with purchasing (2 oz.) and consuming (2 oz.) less SSB. For those who bought ≥1 SSB, the portion size cap was associated with purchasing (11 oz.) and consuming (12 oz.) fewer SSB. No differences in food calories purchased between arenas.
Wickramasekaran et al. (2018) [45]	X			X				Average calories purchased per snack decreased (39%). The average sodium per snack purchased decreased (30%). The average sugar per snack decreased (50%). For beverages, declines were 90%, 25%, and 90%, respectively.
Yan et al. (2019) [67]	X							Declines in saturated fat (0.55 g) and sodium (25 mg) per snack sold. Mean sugar content per beverage sold decreased (12.5 to 13 g) in both settings. Monthly units sold by micro-market: 210 dairy, 85 fruit, and 87 vegetables.
**(c) Key findings from included studies (United States) on diet quality outcomes of HFSG interventions (N = 6)**								
**Author (Year)**	**HFSG Setting(s) ^a^**							**Key Findings: Diet Quality**
	**WS**	**HC**	**M**	**CP**	**HE**	**Rec**	**Ch/Y**	
Belanger and Kwon (2016) [55]			X					After the intervention, soldiers consumed fewer calories overall and from total fat and saturated fat. Intake of sodium and vitamin C was lower. No differences in total cholesterol, iron, fiber, and vitamin A intake.
Berkowitz et al. (2016) [56]	X							Energy intake decreased by 74 kcal (629 to 555) per patron when reduced-size entrees were available compared to baseline. Intakes of total fat, saturated fat, cholesterol, sodium, fiber, calcium, and potassium were also significantly lower.
Cole et al. (2018) [58]			X					The total HEI-2010 score for intervention patrons increased 3 points to reach 60 points over 4–12 mo, compared with the control group, which remained constant. Intervention patrons increased intake of whole fruits, total protein, seafood, and plant protein; decreased intake of vegetables, dairy, and fatty acids; and a significant proportion of patrons’ diet quality moved from “poor” to “needs improvement.”
Crombie et al. (2013) [60]			X					At 6- and 12-month follow-ups, compared to baseline, diners at intervention facilities had significantly lower lunchtime intakes of energy, total fat, percent energy from fat and saturated fat, discretionary fat, and refined grains.
Durant et al. (2020) [61]		X		X			X	Based on difference-in-difference analyses, there were no differences in self-reported consumption of sugary beverages.
Epel et al. (2020) [53]	X	X			X			Overall, less SSB consumption. Participants reported a significant 49% decline (510 mL) in daily SSB intake at 6-month follow-up, which remained stable at 12-month follow-up. There were greater improvements among employees randomized to receive motivational intervention.
**(d) Key findings from included studies (United States) on health outcomes of HFSG interventions (N = 4)**								
**Author (Year)**	**HFSG Setting(s) ^a^**							**Key Findings: Health**
	**WS**	**HC**	**M**	**CP**	**HE**	**Rec**	**Ch/Y**	
Abrahams-Gessel et al. (2022) [42]	X							Within the federal workforce population, the simulated intervention resulted in a lifetime reduction in cases of heart attack, stroke, and diabetes and in deaths from ischemic heart disease and stroke.
Basu et al. (2020) [51]	X	X						Simulated SSB sales ban: reduced the incidence and mortality associated with obesity (−1.0%), coronary heart disease (−2.8%), history of cerebrovascular accidents (−1.3%), diabetes mellitus (−3.2%), chronic kidney disease (−2.5%), dental disease (−3.8%), and other causes of mortality (−2.1%); and accounted for +0.5% in discounted QALYs per 10,000 people, lifetime.
Epel et al. (2020) [53]	X	X			X			Overall, the SSB ban was associated with reductions in both waist circumference and sagittal diameter. No change in body mass index or insulin sensitivity.
Yarnoff et al. (2022) [39]	X	X		X	X		X	If sustained through 2025, intervention was projected to decrease premature deaths (−0.17%) and medical costs (−0.12%) and improve QALYs (+0.77%). If sustained through 2040, projected impacts are greater: −0.19%, −0.15%, and +0.91%, respectively.
**(e) Key findings from included studies (United States) on financial implications of HFSG interventions (N = 13)**								
**Author (Year)**	**HFSG Setting(s) ^a^**							**Key Findings: Financial Implications**
	**WS**	**HC**	**M**	**CP**	**HE**	**Rec**	**Ch/Y**	
Abrahams-Gessel et al. (2022) [42]	X							Within the federal population, the per-person cost of simulated intervention was USD 1.27 (across 5 years) and USD 3.82 (across a lifetime). The intervention was cost-saving across both time periods. Implementation in federal and large private company full-time worker populations over a lifetime would generate USD 752 million in combined total health care cost savings.
Basu et al. (2020) [51]	X	X						Savings of USD 308,949 per 10,000 people over 10 years and USD 706,014 per 10,000 people over their lifetimes due to averted health care and loss of productivity spending. Need a 2.2 oz/person/day SSB reduction to fully offset revenue loss if employees do not switch to buying non-SSB.
Cole et al. (2018) [58]			X					The plate cost increased to USD 14.20/day during the first 6 months of implementation. Between 8 and 12 months of intervention, costs stabilized to USD 12.05–USD 12.95/day (the standard maximum allowance is USD 13.11).
Eneli et al. (2014) [52]	X	X						In cafeteria, food court, and gift and snack shop revenues, annual revenue from total beverage sales increased by 3%. Revenue increased by 19% for all types of milk, 22% for 100% fruit juice, 13% for coffee drinks, and 7% for water. Carbonated beverage sales decreased (−17%). Vending machines had a 22% decrease in beverage sales.
Hansotte et al. (2021) [62]				X				Combined cost savings from reducing sodium content in scalloped and mashed potatoes was USD 0.45/serving.
Jilcott Pitts et al. (2016) [43]	X	X						Despite additional upfront costs from training, labor, and equipment, there were qualitative reports of greater sales and revenue after implementation.
Karpyn et al. (2020) [37]				X				No impact on revenue.
Laroche et al. (2014) [63]							X	Profit margins of healthier items were lower, but average sales per game were higher in 2009 vs. 2008.
Lessard et al. (2014) [64]	X							Compared with the prior year, there was an increased number of total items purchased during the healthy vending pilot. Monthly gains relative to expected profits ranged from 4% to 51% across sites, and monthly losses ranged from −4% to −36%. At one site, profits were greater than expected for 5 of 6 months during the intervention.
Pharis et al. (2017) [49]	X			X		X		Monthly revenues decreased both for snacks (11%) and beverages (21%).
Wickramasekaran et al. (2018) [45]	X			X				Revenue decreased for snacks (37%) and beverages (34%).
Yan et al. (2019) [67]	X							Mean monthly snack revenue increased. Mean monthly beverage revenue increased when sold at the micro-market compared to both pre-policy and vending machine settings.
Yarnoff et al. (2022) [39]	X	X		X	X		X	Three years of implementation: cost USD 10/person reached, USD 42,917 per food service organization. The median monthly cost per modified/substituted food item was USD 684 per food service organization. If sustained through either 2025 or 2040, the Sodium Reduction in Communities Program was estimated to be cost-saving (reduction in medical costs > implementation costs).

HFSG: healthy food service guidelines; SSB: sugar-sweetened beverage; QALY: quality-adjusted life year. Underline indicates primary HFSG intervention setting. ^a^ HFSG settings are abbreviated using WS (worksite), HC (health care), M (military), CP (community programs), HE (higher education), Rec (recreation centers), Ch/Y (child and youth out-of-school programs). Some studies conducted interventions that might overlap with two settings, such as a hospital worksite cafeteria. Because the study population was hospital and health care workers, these studies would be categorized as having occurred in the worksite. Community program settings include congregate meal programs, public zoo facilities, parks, and other food service venues in institutions or programs that serve the public. ^b^ These separate publications evaluated the same HFSG program (Sodium Reduction in Communities Program [SRCP]), so they are presented together as one unique intervention. Long et al. (2021) [29] was a longer-term follow-up to the study initially evaluated in Long et al., 2018 [54]. ^c^ These separate publications evaluated the same HFSG program (Healthy Hospital Food Initiative [HHFI]), so they are presented together as one unique intervention. Moran et al. (2015) [46] assessed non-therapeutic patient menus, while Moran et al. (2016) [30] was a broader evaluation of HHFI in other hospital venues.

## Supplementary Materials

The following supporting information can be downloaded at: https://www.mdpi.com/article/10.3390/ijerph22081194/s1. Table S1: Search strategy for retrieving records for title and abstract screening from the PubMed^®^ database. Table S2: Search strategy for retrieving records for title and abstract screening from the Web of Science database. Table S3: Summary of the distribution in frequency of unique keywords used to describe HFSG publications included for analyses, by retrieval method. Figure S1: Frequency word cloud of all unique keywords (*n* = 118) used to describe healthy food service guideline publications included for analyses that were retrieved through the search strategy. Figure S2: Frequency word cloud of all unique keywords (*n* = 99) used to describe healthy food service guidelines publications included for analyses that were retrieved through snowball sampling.
