# Supplementary material for: Healthy Food Service Guidelines for Worksites and Institutions: A Scoping Review
Source: Int J Environ Res Public Health. 2025 Jul 30;22(8):1194. doi: 10.3390/ijerph22081194 (PMC12386442; doi:10.3390/ijerph22081194)
Supplement: Supplementary file 1 [file ijerph-22-01194-s001.zip › ijerph-3712391-supplementary.pdf]

**Table S1.** Search strategy for retrieving records for title and abstract screening from the PubMed® database.

| No. | Definition | Hits |
|---|---|---|
| #1 | **((((((((((implement*[Title/Abstract]) OR (guideline*[Title/Abstract])) OR (evaluation[Title/Abstract])) OR (impact[Title/Abstract])) OR ("executive order"[Title/Abstract])) OR (intervention[Title/Abstract])) OR (initiative[Title/Abstract])) OR (campaign[Title/Abstract])) OR (procurement[Title/Abstract])) OR (strategy[Title/Abstract])) OR (program[Title/Abstract])) OR (standard*[Title/Abstract])**<br>*Concept 1 (FSG and healthy eating frameworks)* | 5,678,427 |
| #2 | **(((food[Title]) OR (beverage*[Title])) OR (nutrition*[Title])) OR (intake[Title])**<br>*Concept 2 (modifiable food/beverage exposures)* | 276,666 |
| #3 | **((((((((((cafeteria[Title/Abstract]) OR (food service[Title/Abstract])) OR (workplace[Title/Abstract])) OR (work-site[Title/Abstract])) OR (dining[Title/Abstract])) OR (campus[Title/Abstract])) OR (venue*[Title/Abstract])) OR (community[Title/Abstract])) OR (meals[Title/Abstract])) OR (menus[Title/Abstract])) OR (hospital*[Title/Abstract])) OR ((vendor[Title/Abstract]) OR (vending[Title/Abstract]))**<br>*Concept 3 (modifiable environmental exposures)* | 1,774,793 |
| #4 | **#1 AND #2 AND 3**<br>*FSGs and changes to food environment in specific settings* | 13,208 |
| #5 | **(((((((((smoking[Title]) OR (alcohol*[Title])) OR (drug*[Title])) OR (school*[Title])) OR (SNAP[Title])) OR (WIC[Title])) OR (exercise[Title/Abstract])) OR ("physical activity"[Title/Abstract])) OR (Supplemental Nutrition Assistance Program[Title])) OR (microbiome[Title/Abstract])) OR (metaboli*[Title])**<br>*Irrelevant title words* | 1,601,047 |
| #6 | **#4 NOT #5** | 11,193 |
| #7 | **#6**<br>*English language, full-text, 1997-2022* | 8,944 |

**Table S2.** Search strategy for retrieving records for title and abstract screening from the Web of Science database.

| No. | Definition | Hits |
|---|---|---|
| **#1** | **(((TI=(food*)) OR TI=(beverage*)) OR TI=(nutrition)) OR TI=(intake)**<br>*Topic 1 (food, nutrition, environment – modifiable)* | 526,334 |
| **#2** | **((((((((TS=(dining)) OR TS=(military)) OR TS=(cafe*)) OR TS=(work*)) OR TS=(hospital)) OR TS=(campus)) OR TS=(vending)) OR TS=(food service*)) OR TS=(community meals)**<br>*Topic 2 (food settings)* | 7,891,103 |
| **#3** | **((((((TS=(program evaluation)) OR TS=(nutrition standard*)) OR TS=(dietary guideline*)) OR TS=(health promotion)) OR TS=(procurement)) OR TS=(ordinance)) OR TS=(nutrition policy)**<br>*Topic 3 (FSG and related topic words)* | 745,315 |
| **#4** | **((((((((((TS=(SNAP)) OR TS=("Supplemental Nutrition Assistance Program")) OR TS=(WIC)) OR TS=(exercise)) OR TS=(physical activity)) OR TS=(microbiome)) OR TS=(metaboli*)) OR TI=(smoking)) OR TI=(alcohol*)) OR TI=(drug*)) OR TI=(school*)**<br>*Irrelevant topics/outside scope* | 12,110,962 |
| **#5** | **#1 AND #2 AND #3** | 13,437 |
| **#6** | **#5 NOT #4** | 8,989 |
| **#7** | **#6**<br>*Full text, English language, non-MEDLINE, 1997-2022.* | 1,414 |

**Table S3.** Summary of the distribution in frequency of unique keywords used to describe HFSG publications included for analyses, by retrieval method, N (%)

| Frequency[a] | Keywords in search strategy only | Keywords in snowball sampling only | Keywords in both |
|---|---|---|---|
| 1 time | 74 (87.1) | 64 (97.0) | 16 (48.5) |
| 2 times | 7 (8.2) | 2 (3.0) | 7 (21.2) |
| 3-4 times | 3 (3.5) | 0 (0.0) | 7 (21.2) |
| ≥5 times | 1 (1.2) | 0 (0.0) | 3 (9.1) |
| **Total** | 85 (100.0) | 66 (100.0) | 33 (100.0) |

[a] Number of times a keyword was used. Since keywords cannot be re-used within one publication, this number can be interpreted as the number of papers that are partially thematically represented.

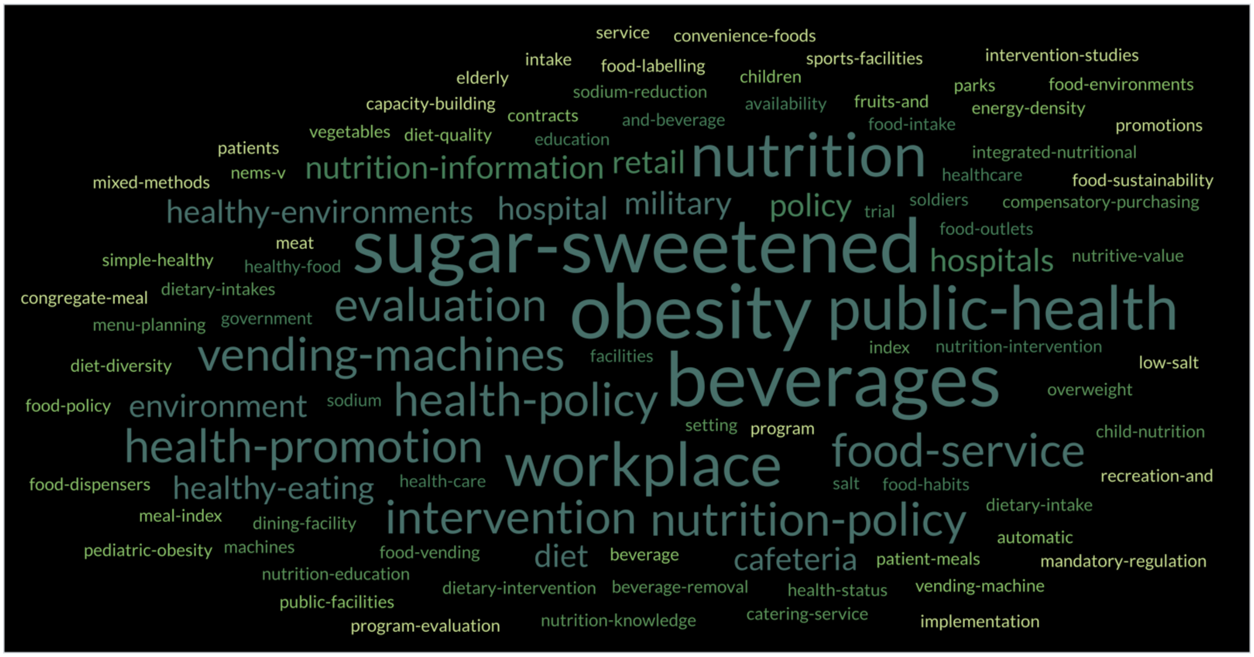


**Figure S1.** Frequency word cloud of all unique keywords (n=118) used to describe healthy food service guidelines publications included for analyses that were retrieved through the search strategy.

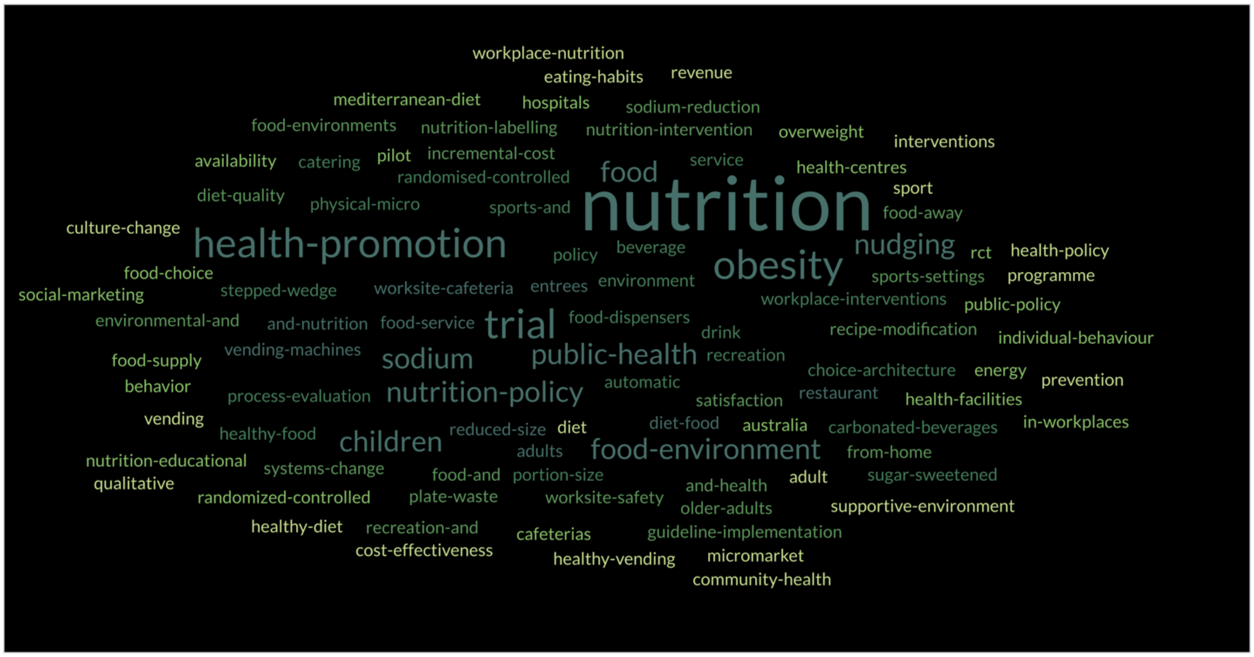


**Figure S2.** Frequency word cloud of all unique keywords (n=99) used to describe healthy food service guidelines publications included for analyses that were retrieved through snowball sampling.
